# Expression Profiles and Prognostic Value of FABPs in Colorectal Adenocarcinomas

**DOI:** 10.3390/biomedicines9101460

**Published:** 2021-10-13

**Authors:** Fidelia Berenice Prayugo, Tzu-Jen Kao, Gangga Anuraga, Hoang Dang Khoa Ta, Jian-Ying Chuang, Li-Chia Lin, Yung-Fu Wu, Chih-Yang Wang, Kuen-Haur Lee

**Affiliations:** 1 International Master/PhD Program in Medicine, College of Medicine, Taipei Medical University, Taipei 11031, Taiwan; m142109005@tmu.edu.tw; 2 Graduate Institute of Cancer Biology and Drug Discovery, College of Medical Science and Technology, Taipei Medical University, Taipei 11031, Taiwan; g.anuraga@unipasby.ac.id (G.A.); d621109004@tmu.edu.tw (H.D.K.T.); m654108001@tmu.edu.tw (L.-C.L.); 3 The PhD Program for Neural Regenerative Medicine, Taipei Medical University, Taipei 11031, Taiwan; geokao@tmu.edu.tw (T.-J.K.); chuangcy@tmu.edu.tw (J.-Y.C.); 4 Research Center of Neuroscience, Taipei Medical University, Taipei 11031, Taiwan; 5 TMU Research Center of Cancer Translational Medicine, Taipei Medical University, Taipei 11031, Taiwan; 6 PhD Program for Cancer Molecular Biology and Drug Discovery, College of Medical Science and Technology, Taipei Medical University and Academia Sinica, Taipei 11031, Taiwan; 7 Department of Statistics, Faculty of Science and Technology, Universitas PGRI Adi Buana, Surabaya 60234, East Java, Indonesia; 8 Cell Physiology and Molecular Image Research Center, Wan Fang Hospital, Taipei Medical University, Taipei 11031, Taiwan; 9 Department of Biomedical Science and Environmental Biology, Kaohsiung Medical University, Kaohsiung 80708, Taiwan; 10 National Defense Medical Center, Department of Medical Research, School of Medicine, Tri-Service General Hospital, Taipei 11490, Taiwan; qrince@yahoo.com.tw; 11 Cancer Center, Wan Fang Hospital, Taipei Medical University, Taipei 11031, Taiwan

**Keywords:** *FABP* family genes, colorectal cancer, *FABP6*, bioinformatics, prognosis

## Abstract

Colorectal cancer (CRC) is one of the world’s leading causes of cancer-related deaths; thus, it is important to detect it as early as possible. Obesity is thought to be linked to a large rise in the CRC incidence as a result of bad dietary choices, such as a high intake of animal fats. Fatty acid-binding proteins (FABPs) are a set of molecules that coordinate intracellular lipid responses and are highly associated with metabolism and inflammatory pathways. There are nine types of *FABP* genes that have been found in mammals, which are *FABP1–7*, *FABP9*, and *FABP12*. Each *FABP* gene has its own roles in different organs of the body; hence, each one has different expression levels in different cancers. The roles of *FABP* family genes in the development of CRC are still poorly understood. We used a bioinformatics approach to examine *FABP* family gene expression profiles using the Oncomine, GEPIA, PrognoScan, STRING, cBioPortal, MetaCore, and TIMER platforms. Results showed that the *FABP6* messenger (m)RNA level is overexpressed in CRC cells compared to normal cells. The overexpression of *FABP6* was found to be related to poor prognosis in CRC patients’ overall survival. The immunohistochemical results in the Human Protein Atlas showed that FABP1 and FABP6 exhibited strong staining in CRC tissues. An enrichment analysis showed that high expression of *FABP6* was significantly correlated with the role of microRNAs in cell proliferation in the development of CRC through the insulin-like growth factor (IGF) signaling pathway. *FABP6* functions as an intracellular bile-acid transporter in the ileal epithelium. We looked at *FABP6* expression in CRC since bile acids are important in the carcinogenesis of CRC. In conclusion, high *FABP6* expression is expected to be a potential biomarker for detecting CRC at the early stage.

## 1. Introduction

Colorectal cancer (CRC) is the third most prevalent malignant tumor in the world and the second largest cause of cancer-related deaths. In 2020, there were 1,148,515 and 732,210 new instances of colon and rectal cancers reported worldwide, respectively, and it is anticipated that more than half of colon and rectal cancer patients will die from this condition [1]. The large rise in CRC incidence in both industrialized and developing nations is thought to be linked to population aging, bad dietary choices (e.g., excessive animal fat intake and insufficient cellulose intake), lack of physical activity, obesity, and smoking [2].

Fatty-acid binding proteins (FABPs) are a class of molecules that help cells organize their lipid responses, and they are also involved in metabolic and inflammatory processes [3]. The *FABP* genes family encodes transcription factors that binds free FAs, cholesterol, and retinoids [4,5]. Some FABP polymorphisms have been linked to lipid metabolism problems and the onset of atherosclerosis, such that FABP levels in the blood are utilized as indicators of tissue injury [6]. Since the discovery of 12 different forms of *FABP* (*FABP1–12*), nine of them (*FABP1–FABP9*) have been verified to be expressed in humans [7]. According to the available literature, *FABP*s are expressed differently in various kinds of cancer or different cell lines of the same disease [8]. In a broader context, *FABP*s are lipid chaperones that transport and regulate the biological activities of lipids. Variations in *FABP* expression may contribute to tumor development in humans throughout tumor progression. *FABP3* [9] in gastric cancer, *FABP5* [10] and *FABP9* in prostate cancer [11], and *FABP7* in clear-cell renal cell carcinoma [12] have recently been discovered to be diagnostic markers and/or novel therapeutic targets in various cancers.

FABP6 is a bile-acid intracellular transporter in ileal epithelial cells, which supports catalytic cholesterol and metabolism; it is also known as ileal bile-acid-binding protein (*I-*BABP) and gastrotropine. Bile-acid concentrations, particularly secondary bile acids, are known to be higher in colon adenomas, whereas bile acids promote enhanced *FABP6* expression in colon cancer cell lines in vitro [13]. Fujii et al. found that *FABP6* expression was due to bile-acid exposure in a colon cancer cell line [14]. Excess bile acids, especially secondary bile acids, which enter epithelial cells at variable quantities, cause apoptosis and DNA damage, resulting in genetic changes in the colonic epithelium [15]. Furthermore, unusually high bile-acid levels, which connect the intestinal microbiota with the liver and intestinal metabolism, have a substantial detrimental influence on the colonic mucosa, hastening the course of CRC [16,17]. In the early stage of CRC, *FABP6* was recognized as an overexpressed gene [18]. It is important to design appropriate and efficient early diagnoses and new therapeutic targets. Thus, it is crucial to detect new biomarkers which can provide a fresh understanding of the molecular abnormalities that lead to CRC. Although many researchers have worked hard to understand the mechanisms of *FABP6* in CRC regulation, more research is needed [19,20,21].

In our study, we performed multi-omics analyses to investigate the expression of *FABP* genes in human CRC. Expression, mutation, and alteration of the *FABP* genes were evaluated using the Oncomine, CCLE, GEPIA, and cBioPortal platforms, and the correlation between these *FABP* members and clinical outcomes in CRC were examined using PrognoScan and the Human Protein Atlas database analyses [22,23,24,25,26,27]. The protein–protein interactions (PPIs) between each FABP family member and its predicted functional partner were explored using STRING, whereas we used the TIMER database to identify potential biomarkers in immune infiltration. Additionally, the genes commonly correlated with the *FABP* genes were categorized in key pathways related to the tumorigenesis of CRC and analyzed using MetaCore [28,29,30,31,32]. The present study suggests the prognostic value of the *FABP6* gene expression and its associated pathways in human CRC. Therefore, *FABP6* is expected to be a candidate biomarker in the diagnosis and prognosis of colorectal adenocarcinomas (COADs).

## 2. Materials and Methods

### 2.1. Data Collection

mRNA sequencing data, molecular categories, immunohistochemistry (IHC) staining data, and clinical information of colorectal cancer patients and other cancer types were obtained from the TCGA COAD and READ databases (https://tcga-data.nci.nih.gov/, accessed on 14 May 2021), Human Protein Atlas (https://www.proteinatlas.org/, accessed on 14 May 2021), and GEPIA (http://gepia.cancer-pku.cn/index.html, accessed on 14 May 2021), respectively. The expression pattern of normal colon tissues and tumor tissues was acquired from the TCGA database. The STRING (https://www.string-db.org/, accessed on 14 May 2021) database and MetaCore (https://portal.genego.com/, accessed on 14 May 2021) platform were used to construct the PPI network and enrichment pathway. We obtained the immune cell content file of the TCGA sample from the TIMER database (https://cistrome.shinyapps.io/timer/, accessed on 14 May 2021).

### 2.2. Expression Analysis of FABPs in CRC

To reveal the gene expressions of *FABP* genes, we performed exploratory analyses using the Oncomine (https://www.oncomine.org accessed on 14 May 2021) [33] and GEPIA2 databases (http://gepia2.cancer-pku.cn/#index accessed on 14 May 2021) [34]. Oncomine is a platform consisting of 715 datasets containing 86,733 samples. This study used a set threshold for the interrogation of *FABP* gene expression profiles. To confirm differences in expressions across 20 different cancers, we used a significant *p*-value of <0.05, a multiple of change of 1.5, and a gene rank in the top 10%. We used the CCLE dataset (https://portals.broadinstitute.org/ccle accessed on 14 May 2021) to further examine the messenger (m)RNA expression levels of *FABP*s in cancer cell lines, and the expression data are displayed in heatmaps [35]. In this work, we also utilized GEPIA2 to look at the gene expression patterns of *FABP* genes in normal and CRC tissues. To evaluate paired tumor vs. normal samples, an analysis of variance (ANOVA) was performed, with the threshold absolute log base 2 of the fold change (Log_2_FC) set to 1 and the value of *q* set to 0.05 [36,37,38,39,40,41,42,43].

### 2.3. Prognostics and Protein Expression Analysis of FABPs in CRC

The distribution of FABP proteins in CRC tissues in the human body were investigated using the Human Protein Atlas (HPA) database (https://www.proteinatlas.org/ accessed 14 May 2021). More than 400,000 high-resolution pictures matched to more than 700 antibodies to human proteins are available in the HPA database [44,45]. Each intensity and fraction combination is automatically converted into a protein expression level score using the following formula: negative—not detected; weak—not detected; weak combined with either 25–75 percent or 75 percent—low; moderate—low; moderate combined with either 25–75 percent or 75 percent—medium; strong—medium, strong combined with either 25–75 percent or 75 percent—high. Furthermore, we also used the PrognoScan database to find correlations between *FABP* genes and patient prognoses in overall survival (OS). The PrognoScan database is a meta-analysis database that is used to show a gene’s predictive value [46].

### 2.4. Functional Enrichment Analysis of FABPs in CRC

To study the list of classes of genes or proteins in terms of biological functions which may have a relationship with a disease phenotype, this research used an integrative analysis involving several databases such as STRING, cBioPortal, and MetaCore [47,48,49,50,51,52]. The STRING database (https://www.string-db.org/ accessed on 14 May 2021), consisting of 24,584,628 proteins from 5090 organisms, was used to reveal protein–protein interactions (PPIs) [53,54]. Meanwhile, to study interconnections between *FABP* genes, we used the cBioPortal database, results of which we then displayed in correlation plots [55,56]. Lastly, we used the MetaCore (https://portal.genego.com/ accessed on 14 May 2021) platform to more deeply reveal the signaling pathways and biological processes of *FABP* genes in CRC [57,58,59,60,61,62,63].

### 2.5. TIMER Analysis of FABPs in CRC

TIMER (http://timer.cistrome.org/ accessed on 14 May 2021) is a comprehensive database that provides analysis of immune infiltrates in various types of cancer [64,65,66]. We utilized this database to uncover the involvement of *FABP* genes in immune infiltrates in CRC. Through scatterplots, we present the relationships between the estimated infiltrate values and gene expressions levels, and we evaluated the significance level of the correlations at *p* < 0.05.

## 3. Results

### 3.1. Expression Analysis of FABPs in CRC Using Oncomine and GEPIA2

**Figure 1:**
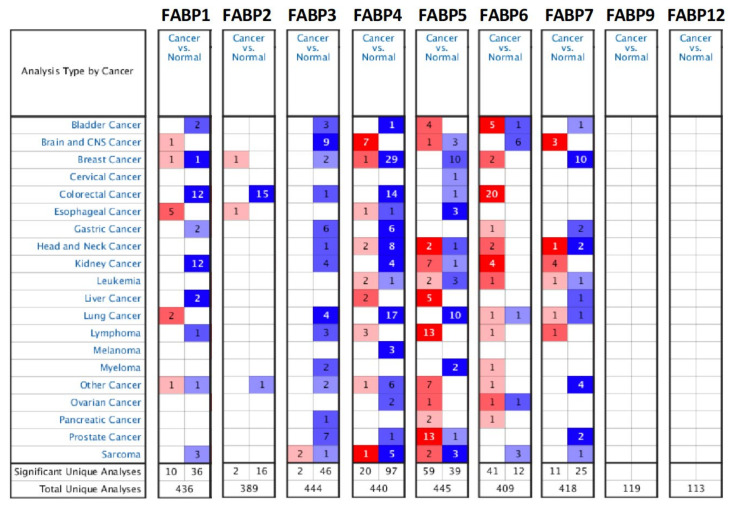
The mRNA expression study of fatty acid-binding proteins (*FABP*s) in normal and CRC tissues (Oncomine database). Color gradients represent gene ranking percentiles; a significant level was accepted at *p* < 0.05, a multiple of change of 1.5, and a gene ranking in the top 10%.

**Figure 2:**
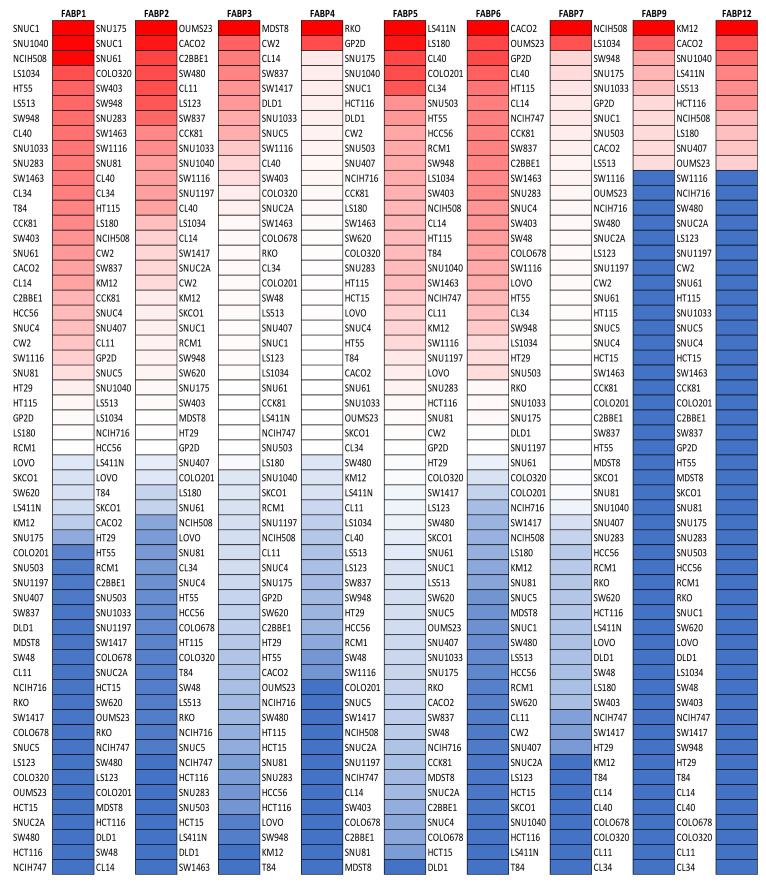
Heatmap of fatty acid-binding protein (*FABP*) gene mRNA expressions in colorectal cancer cell lines (CCLEs), with red signifying overexpression and blue suggesting underexpression.

Oncomine analysis was performed to evaluate the *FABP* gene family to understand the expression profiles of the *FABP* gene family in different cancer types. As shown in Figure 1, there were 20 studies that examined high mRNA expression levels of *FABP6* in CRC tissues, to which we assigned a significance level of *p* < 0.05, multiple of change of 1.5, and a gene rank in the top 10%. Next, the mRNA expression levels of *FABP* family members in CRC cell lines are also presented using CCLE analysis (Figure 1). The high mRNA expression of *FABP*s in some cell lines is indicated by the red color in the heatmap shown in Figure 2.

**Figure 3:**
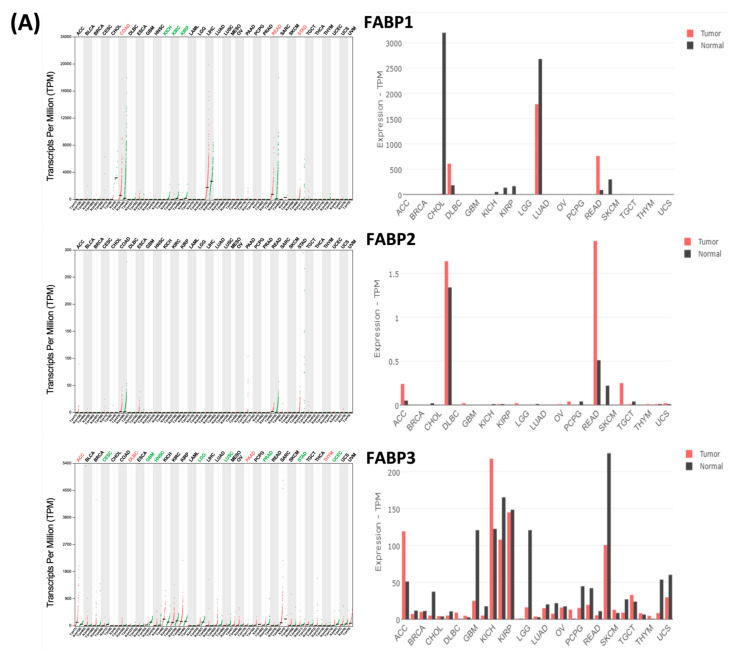

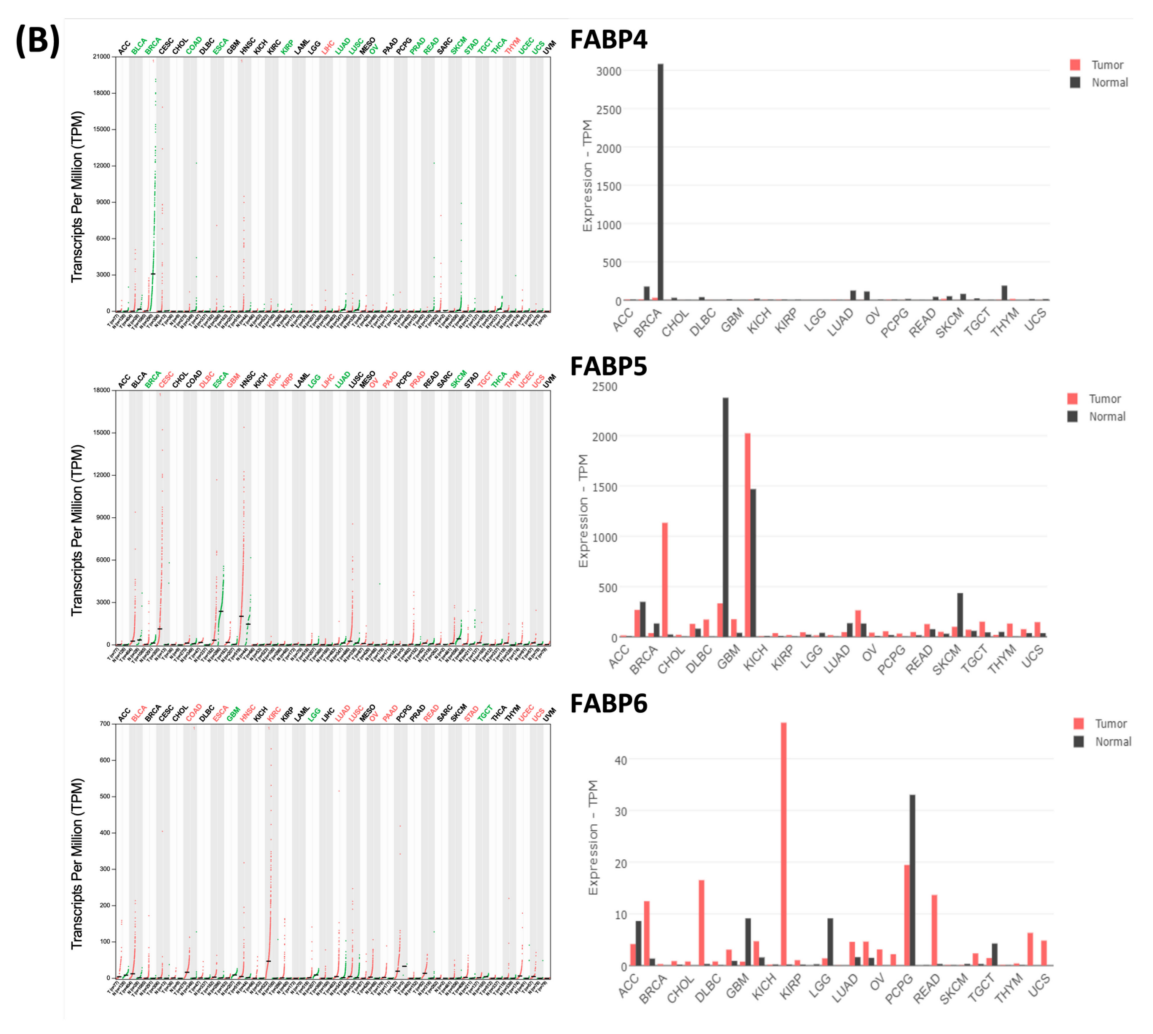

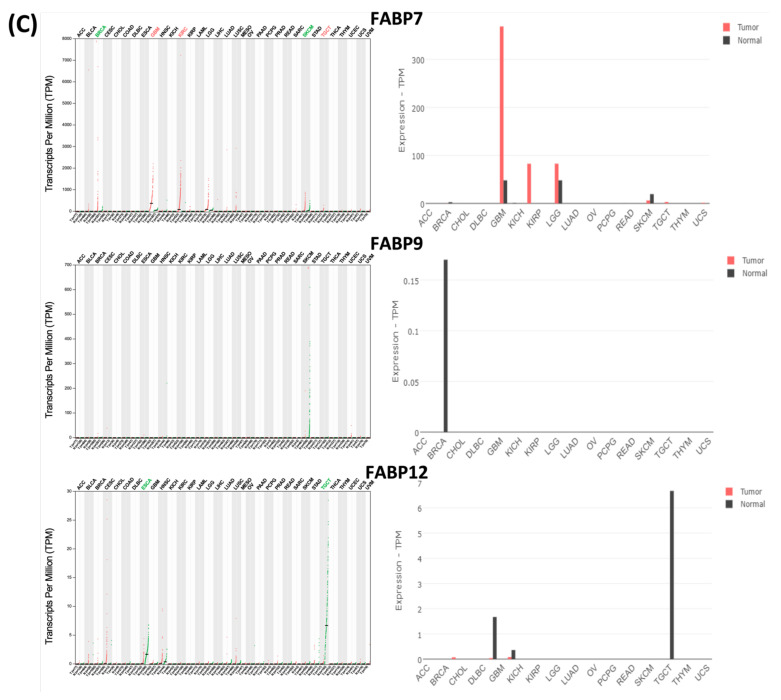
The gene expression profile of fatty acid-binding proteins (*FABP*s) (**A**) FABP1, FABP2 and FABP3; (**B**) FABP4, FABP5 and FABP6; (**C**) FABP7, FABP9 and FABP12 across 27 TCGA tumor samples and paired normal tissues using GEPIA (Gene Expression Profiling Interactive Analysis) webserver. The red bar indicates the expression of *FABP* genes in tumor tissues, while black indicates the expression in normal tissues. For each TCGA tumor (red), its matched normal and GTEx data (green) are given; T: tumor; N: normal; n: number. Y axis: transcript per million (log2(TPM + 1)). X axis: number of tumor and normal samples. ACC, adrenocortical carcinoma; BLCA, bladder urothelial carcinoma; BRCA, breast invasive carcinoma; CESC, cervical squamous cell carcinoma and endocervical adenocarcinoma; CHOL, cholangiocarcinoma; COAD, colon adenocarcinoma; DLBC, lymphoid neoplasm diffuse large B-cell lymphoma; ESCA, esophageal carcinoma; GBM, glioblastoma multiforme; HNSC, head and neck squamous cell carcinoma; KICH, kidney chromophobe; KIRC, kidney renal clear cell carcinoma; KIRP, kidney renal papillary cell carcinoma; LAML, acute myeloid leukemia; LGG, brain lower-grade glioma; LIHC, liver hepatocellular carcinoma; LUAD, lung adenocarcinoma; LUSC, lung squamous cell carcinoma; MESO, mesothelioma; OV, ovarian serous cystadenocarcinoma; PAAD, pancreatic adenocarcinoma; PCPG, pheochromocytoma and paraganglioma; PRAD, prostate adenocarcinoma; READ, rectum adenocarcinoma; SARC, sarcoma; SKCM, skin cutaneous melanoma; STAD, stomach adenocarcinoma; TGCT, testicular germ cell tumors; THCA, thyroid carcinoma; THYM, thymoma; UCEC, uterine corpus endometrial carcinoma; UCS, uterine carcinosarcoma.

**Figure 4:**
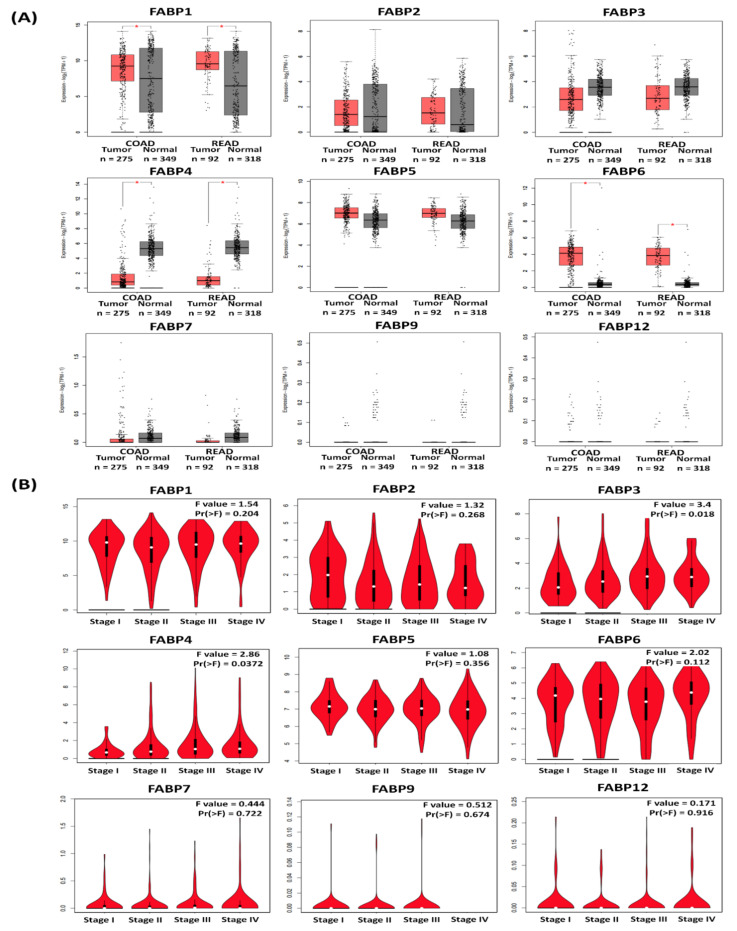
Gene expression profile of the fatty acid-binding proteins (*FABP*s) in CRC. (**A**) Gene expression analysis of *FABP*s genes using GEPIA based on TCGA database. Boxplot of transcriptional levels of *FABP*s in terms of log_2_(TPM + 1) in COAD (*n* = 275) vs. normal samples (*n* = 349) and READ (*n* = 92) vs. normal samples (*n* = 318). Red indicates the *FABP* genes transcriptional levels in COAD and READ tissues, while black indicates the transcriptional levels in normal tissues. The method for differential analysis was one-way ANOVA. Statistical significance is indicated by *p* < 0.05. (**B**) Gene expression analysis across the stages of the *FABP*s genes in colorectal cancer. The violin plot shows comparisons of the expressions of the *FABP* genes family from TCGA data in CRC during cancer progression. Violin plots represent log_2_(TPM + 1) of genes in stage 1 CRC, stage 2 CRC, stage 3 CRC, and stage 4 CRC. An independent *t*-test was used to calculate *p*-values; *p* < 0.05 was considered statistically significant; *Pr(>F)* < 0.05, based on Student’s *t*-test. Values are the mean ± SEM. TPM, transcript per million.

Next, to further examine the expression levels of *FABP* family members in CRC, we performed a GEPIA2 analysis. According to the analytical results from GEPIA2 (Figure 3 and Figure 4), the *FABP1* and *FABP6* genes had different and significant expressions in CRC (Figure 3A,B). Meanwhile, *FABP2*, *FABP3*, *FABP4*, *FABP5*, *FABP7*, *FABP9*, and *FABP12* exhibited no significant differences in CRC (Figure 3). We also present in boxplot visualizations comparisons of the expressions of *FABP* genes in normal, colon adenocarcinoma (COAD), and rectal adenocarcinoma (READ) tissues (Figure 4), which showed that the *FABP1*, *FABP4*, and *FABP6* genes had significantly different expression levels in normal and CRC tissues (Figure 4A). Furthermore, the association of expression levels of *FABP* family members with clinicopathological parameters in CRC patients was investigated. As shown in Figure 4B, we found that the mRNA expression levels of *FABP3* or *FABP4* were related to different tumor stages of CRC. However, mRNA expression levels of *FABP1, FABP2, FABP5, FABP6, FABP7, FABP9*, or *FABP12* did not significantly differ in different tumor stages in CRC patients. This result is consistent with a previous study which also showed that *FABP6* may play an important role in early carcinogenesis, but not in cancer stage progression in CRC [18].

### 3.2. Prognostic Value and Protein Expression Analysis of FABPs in CRC

**Figure 5:**
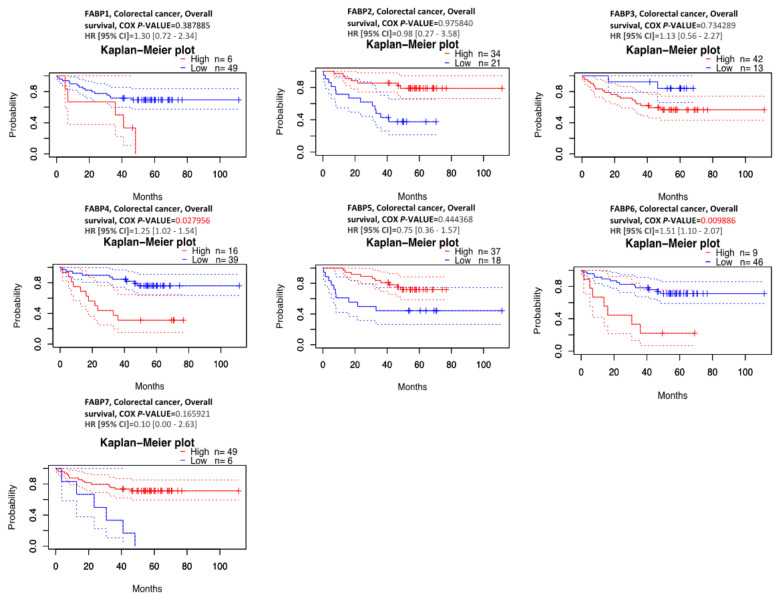
Kaplan–Meier plot of fatty acid-binding protein (*FABP*) expression in CRC patients (*n* = 55) for overall survival analysis (PrognoScan database). The survival curves for the high (red) and low (blue) expression groups are dichotomized at the optimum cutpoint. The time on the X-axis corresponds to the survival rate on the Y-axis. Dotted lines also show the 95 percent confidence intervals for each group. The hazard ratio (HR) is a relative prognostic measure of patients with CRC. Log (rank *p*) was used to determine the level of prognostic significance of patients with CRC. Furthermore, log (rank *p* < 0.05) was interpreted as a significant difference in the prognostic expression of patients with CRC. The prognostic value of *FABP4* and *FABP6* showed significance in terms of overall survival outcomes in CRC (Cox *p* < 0.05).

This paper presents an examination of CRC patient survival using a survival curve to assess the predictive relevance of *FABP* family member expression levels in predicting survival outcomes of CRC patients. Survival statistics of 55 CRC patients, as well as the hazard ratios (HR) and *p*-values for statistical significance are shown in Figure 5. Regarding overall survival (OS), we found that *FABP4* (Cox *p* = 0.027956, hazard ratio (HR) (95% confidence interval (CI)) = 1.25 (1.02–1.54)) and *FABP6* (Cox *p* = 0.009886, HR (95% CI) = 1.51 (1.10–2.07)) were significantly correlated with the prognosis of CRC patients. *FABP1* (Cox *p* = 0.387885, HR (95% CI) = 1.30 (0.72–2.34)), *FABP2* (Cox *p* = 0.975840, HR (95% CI) = 0.98 (0.27–3.58)), *FABP3* (Cox *p* = 0.734289, HR (95% CI) = 1.13 (0.56–2.27)), *FABP5* (Cox *p* = 0.444368, HR (95% CI) = 0.75 (0.36–1.57)), and *FABP7* (Cox *p* = 0.165921, HR (95% CI) = 0.10 (0.00–2.63)) were not significantly correlated with the prognosis of CRC patients.

**Figure 6:**
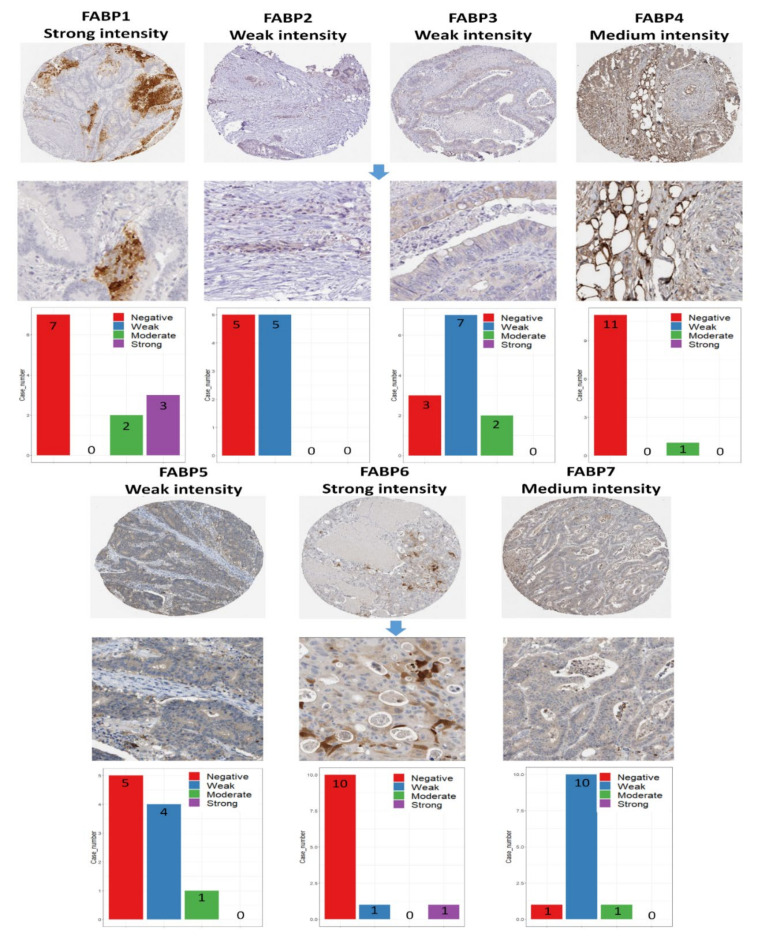
Analysis of protein expression using the HPA database in CRC tissue samples. The representative images of immunohistochemistry of *FABP* genes show their staining intensity, and the bar charts represent the quantification of IHC staining in CRC samples. Manual scoring of immunohistochemistry data for staining intensity (negative, weak, moderate, or strong) and proportion of stained cells (25 percent, 25–75 percent, or >75 percent) determined the protein expression score. *FABP1* (11 patients), *FABP2* (10 patients), *FABP3* (12 patients), *FABP4* (12 patients), *FABP5* (10 patients), *FABP6* (12 patients), and *FABP7* (12 patients). *FABP1* and *FABP6* showed strong protein expressions in CRC, whereas *FABP2, FABP3, FABP4, FABP5*, and *FABP7* showed low protein expressions in CRC.

Next, we validated the protein expression levels of *FABP* family members in CRC tissues using the Human Protein Atlas (HPA) database. The representative IHC images of immunoreactivity expression in cancer specimens (Figure 6, Table S1) are shown. Manual scoring of immunohistochemistry data for staining intensity (negative, weak, moderate, or strong) and proportion of stained cells (25 percent, 25–75 percent, or >75 percent) determined the protein expression score. The protein expression levels of *FABP1* and *FABP6* were higher in CRC tissues. Overall, *FABP4* and *FABP7* showed a moderate staining intensities, and we already knew that *FABP4* has a role in the early development of CRC. However, since *FABP6* showed strong intensity in IHC staining, we considered *FABP6* to be a more-promising biomarker of CRC development. On the other hand, *FABP2, FABP3*, and *FABP5* were underexpressed in CRC specimens.

### 3.3. Gene Correlation Analysis and Protein–Protein Interactions (PPIs) of FABPs in CRC

**Figure 7:**
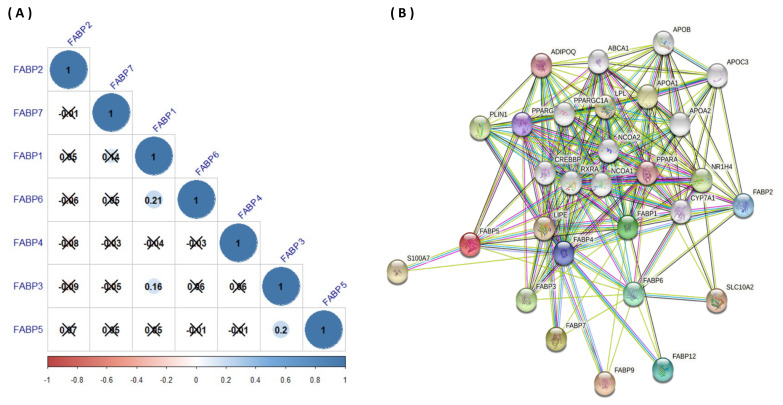
(**A**) Correlation analysis of fatty acid-binding protein (*FABP*) genes in CRC (cBioportal database). Correlation plots among *FABP* family genes in CRC. Insignificant correlation values are indicated by crosses; *p* < 0.05 was considered statistically significant. (**B**) Protein–protein interactions (PPIs) of *FABP*s family members (STRING database). Highly interacting proteins are represented as hub protein nodes in the PPI network. The nodes also show the known or anticipated three-dimensional protein structures. Proteins involved in cell death control are represented by red nodes. Proteins involved in the immune system are represented by green nodes. Proteins involved in neutrophil degranulation are represented by blue nodes. Proteins involved in RNA metabolism are represented by pink nodes. Proteins linked to chromatin DNA binding are represented by yellow nodes. The types of evidence supporting these hypothesized connections are shown by lines of different colors.

To further explore the gene–gene interaction network and potential regulation of *FABP*s in CRC, we performed data mining and constructed an interaction network using cBioPortal and STRING online software. Based on the correlation analysis among *FABP* genes, we found that *FABP1* was significantly and positively correlated with *FABP6* using the cBioPortal database analysis (Figure 7A). According to above-mentioned results, the HPA database pointed out that not only did FABP6 protein expression show a strong intensity of staining, but also the FABP1 protein was highly expressed in CRC tissues, suggesting their potential correlation. In addition, *FABP1* was also positively correlated with *FABP3*, and *FABP3* was positively correlated with *FABP5*. Thus, a PPI network analysis was performed for FABP transport proteins using a STRING analysis. The PPI network yielded a number of edges and nine nodes. The impacts of the nine FABP transport proteins were largely related to fatty acid (FA) receptor binding and activity; in addition, FABP1 was demonstrated to increase free fatty acids (FFAs) absorption into the cytoplasm and facilitate transport to the nucleus, with a preference for longer-chain FAs [67]. The STRING database was also utilized to look at the top ten most often interacting adjacent proteins among the nine FABP family members. PPARG, ADIPOQ, PPARA, SLC10A2, LIPE, LPL, S100A7, APOA1, PLIN1, and NR1H4 were shown to be highly associated with FABP transport protein modulation and capacity in CRC patients (Figure 7B). SLC10A2 is a sodium/bile-acid cotransporter found in the ileum that is involved in the sodium-dependent reabsorption of bile acids from the small intestine lumen, as well as cholesterol metabolism [68]. Nuclear receptor subfamily 1 group H member 4 (NR1H4), also known as farnesoid X receptor (FXR), is a bile-acid receptor that promotes transcriptional activation of *FABP6* as one of its target genes [69,70]. It was found that expression of NR1H4 is frequently downregulated by DNA methylation and Kirsten rat sarcoma (KRAS) signaling in colon cancer, which implies a negative relationship between NR1H4 and tumor growth [71].

### 3.4. Functional Enrichment Analysis of FABPs Using the MetaCore Platform

**Figure 8:**
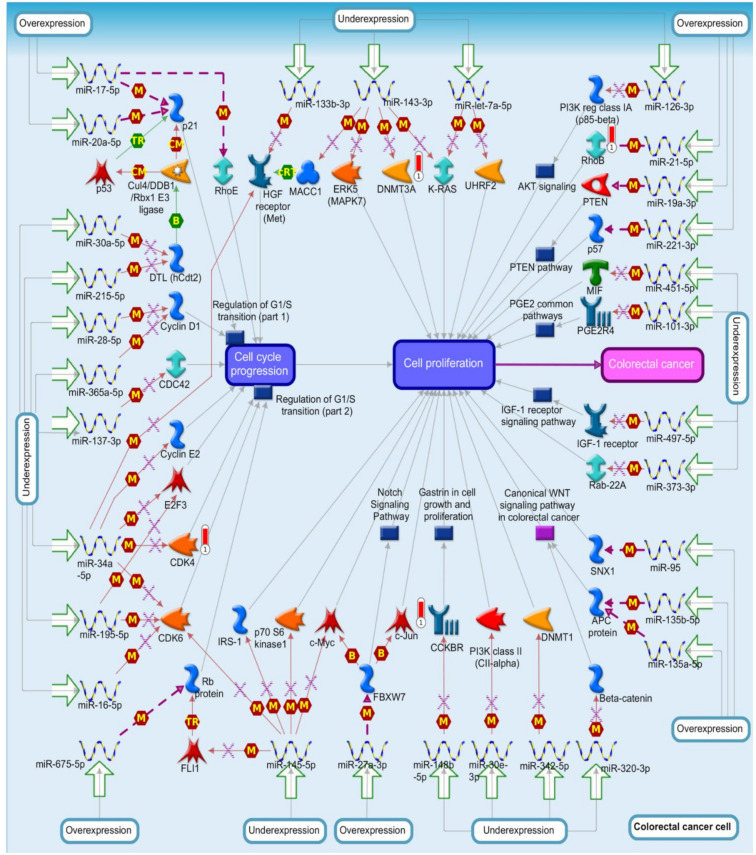
MetaCore pathway analysis of fatty-acid binding protein 6 (*FABP6*) co-expressed differentially expressed genes (DEGs) in colorectal cancer patient databases. *FABP6* co-expressed genes in colorectal cancer from TCGA databases were acquired and identified by Venn diagram analysis. These 243 genes were further exported to the MetaCore pathway analysis tool to identify gene networks and signaling pathways. The “role of microRNAs in cell proliferation in CRC” was identified as one of the top three enriched pathways in the formation of CRC. The network suggested that the differential expression of microRNAs will lead to colorectal cancer cell progression. B, binding; CM, covalent modification; +*p*, phosphorylation; T, transformation; Tn, transport; Z, catalysis; TR, transcription regulation; IE, influence on expression; GR, group relation; CS, complex subunit. A green arrow represents (positive) activation of the process. A red arrow represents (negative) inhibition of the process. A gray arrow represents an unspecified process.

MetaCore has different biomolecular interactions revealing relevant signal pathways such as protein–protein, protein–DNA, and protein–metabolite connections. To reveal deeper biological processes, we leveraged the MetaCore platform by uploading co-expressed *FABP* gene data from The Cancer Genome Atlas (TCGA)-CRC dataset. The top 10% differentially expressed genes (DEGs) were submitted to MetaCore for paired CRC cancer samples and matched normal tissues. Analysis of biological processes revealed that the *FABP1* gene had significant involvement in “recurrent gene fusions in prostate cancer”, “transcription_*FXR*-regulated cholesterol and bile-acid cellular transport”, and “transcription_*HIF-1* targets” in CRC development (Figure S2, Table S2). This result confirms that *FABP1* is positively correlated with FABP6, as we previously mentioned in the results of the gene correlation analysis. *FABP1* has a role in bile-acid cellular transport regulated by *FXR* or *NR1H4*. Results of the transcriptome analysis revealed that the weaning transition changed the expression of genes involved in food transport and metabolism in the intestine. Weaning reduced the production of *FABP1* in the gut, which is important for fatty acid (FA) metabolism. Weaning enhanced the expression of genes involved in bile-acid metabolism, such as *FABP6* and *FXR* or *NR1H4* [72]. *FABP2* was significantly correlated with “estradiol metabolism”, “retinol metabolism”, and “*PXR*-mediated direct regulation of xenobiotic metabolizing enzymes/rodent version” in the development of CRC (Figure S3, Table S3). *FABP3* was significantly correlated with “CHDI_correlations from replication data_causal network (positive correlations)”, “rheumatoid arthritis (general schema)”, and “immune response_immunological synapse formation” in the development of CRC (Figure S4, Table S4). *FABP4* was significantly correlated with “immune response_lectin-induced complement pathway”, “immune response_classical complement pathway”, and “COVID-19-associated coagulopathy” in the development of CRC (Figure S5, Table S5). FABP5 was significantly correlated with “DNA damage_intra S-phase checkpoint”, “cell cycle_role of APC in cell-cycle regulation”, and “DNA damage_role of Brca1 and Brca2 in DNA repair” in CRC development (Figure S6, Table S6). *FABP6* was significantly correlated with “ubiquinone metabolism”, “l-lysine metabolism”, and “role of microRNAs in cell proliferation in colorectal cancer” in the development of CRC (Figure 8, Table S7). Discrepancies in the expressions of some genes were confirmed to be the cause of several human malignancies. In addition, several CRC patients with tumors of a histologically identical type had uneven responses to treatment and idiosyncratic symptoms. Micro (mi)RNAs have critical roles in a variety of biological functions, including cancer cell proliferation, invasion, and metastasis [73,74]. Some previous studies on miRNAs and hub genes involved in CRC identified 10 hub genes, *SLC26A3, CLCA1, CLCA4, GUCA2A, GUCA2B, MS4A12, KRT20, AQP8, MAOA,* and *ADH1A*, four differentially expressed miRNAs, miR-423-5p, miR-552, miR-502-3p, and miR-490-5p [75], and a specific miRNA and its role in CRC by targeting specific hub genes [76,77,78,79]. *FABP7* was significantly correlated with “breakdown of CD4^+^ T-cell peripheral tolerance in type 1 diabetes mellitus”, “immune response_IL-12 signaling pathway”, and “immune response_T-cell co-signaling receptors, schema” in CRC development (Figure S7, Table S8). *FABP9* was significantly correlated with “putative role of Tregs in COPD”, “breakdown of CD4^+^ T-cell peripheral tolerance in type 1 diabetes mellitus”, and “immune response_IL-16 signaling pathway” in the development of CRC (Figure S8, Table S9). FABP12 was significantly correlated with “protein folding and maturation_posttranslational processing of neuroendocrine peptides”, “translation_regulation of EIF2 activity”, and “development_TGF-beta-dependent induction of EMT via MAPK” in CRC development (Figure S9, Table S10).

### 3.5. Associations of FABP Gene Family Members with Immune Cell Infiltration

**Figure 9:**
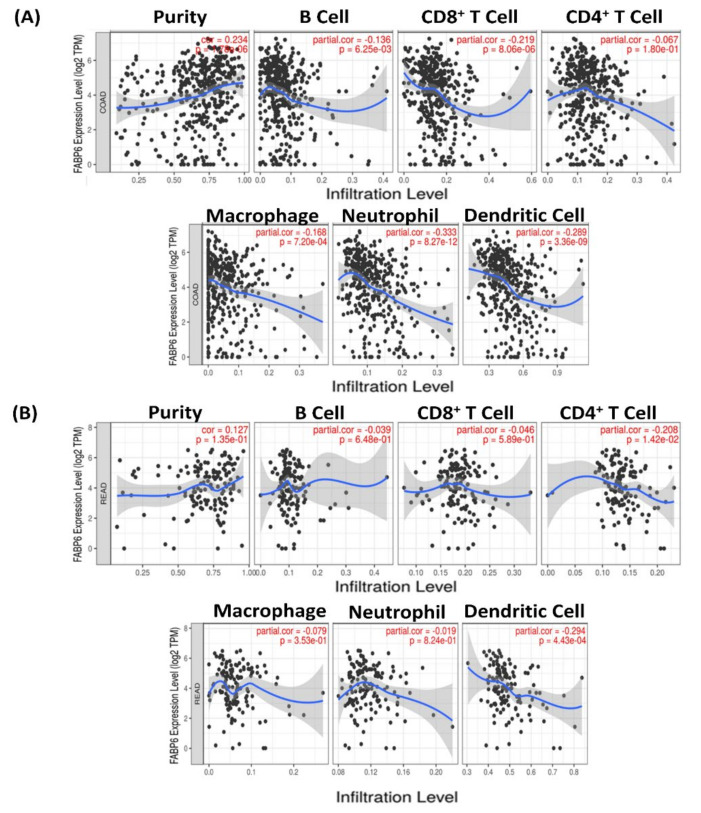
Correlations between differentially expressed fatty acid-binding protein 6 (*FABP6*) gene and immune cell infiltration in primary (**A**) colon adenocarcinoma (COAD) and (**B**) rectal adenocarcinoma (READ) patients. Analysis of the correlation between *FABP6* gene overexpression and some tumor infiltration parameters in both COAD and READ patients was accomplished using TIMER. The figure shows that the *FABP6* gene expression was associated with tumor purity and several tumor-infiltrating immune cell markers, such as CD8^+^ T cells, B cells, CD4^+^ T cells, neutrophils, macrophages, and dendritic cells. Spearman correlations were applied to describe correlations between *FABP* family genes and the abovementioned immune cells (*p* < 0.05 was accepted as statistically significant).

In CRC patients, clinical outcomes are linked to the inflammatory response and immune cell infiltration, both of which are influenced by *FABP* family genes [80]. The TIMER database was used to comprehensively search to see whether *FABP* gene expressions were linked to immune infiltration in CRC patients (Figure 9 and Figure S1A–F). Analytical results showed a correlation between *FABP6* and cluster of differentiation 4^+^ (CD4^+^) T cell, while macrophages and neutrophils were negatively correlated in COAD and READ patients (Figure 9). *FABP1* was negatively correlated with CD4^+^ T cells and neutrophils in COAD and READ patients (Figure S1A). *FABP2* was negatively correlated with B cells in COAD and READ patients (Figure S1B). *FABP3* enhanced the infiltration of B cells and macrophages in READ patients and CD4^+^ in COAD patients (Figure S1C). There was a correlation between *FABP4* and CD4^+^ T cells, while macrophages and neutrophils were definitely corelated in COAD and READ patients (Figure S1D). *FABP5* was negatively correlated with B cells, macrophages, and dendritic cells in COAD patients (Figure S1E). In COAD and READ patients, there was no obvious linear correlation between *FABP7* and immune cell infiltration (Figure S1F).

## 4. Discussion

FABPs are a group of FA transport proteins. FABPs are tiny (12–15 kDa) cytosolic proteins that are abundant in organs with active lipid metabolism, such as the heart and liver, as well as cell types specialized in lipid storage, trafficking, and signaling, such as adipocytes and macrophages. [81]. We explored mRNA expression levels, correlations with clinicopathologic parameters, and prognostic values of nine *FABP* genes in patients with CRC. The majority of these genes had altered expressions that could impact the survival of patients with cancer. *FABP6* might be a viable therapeutic target for colorectal cancer. *FABP6* expression was enhanced in CRC tissues compared to normal tissues, which is consistent with a recent study that found increased expression of this *FABP* in CRC [18]. We discovered that elevated *FABP4* and *FABP6* expressions were linked to a poor prognosis in CRC patients. In addition, we found that *FABP6* was significantly correlated with the “role of microRNAs in cell proliferation in colorectal cancer” in the development of CRC using MetaCore analysis. In the present study, our main focus was on *FABP6* because its expression is significantly overexpressed in CRC. In our previous study of prostate cancer, we discovered that miR-320 inhibited the canonical *Wnt*/β-catenin pathway, which means this miRNA plays a role in malignant transformation and cancer initiation [82]. Activation of the *Wnt*/β-catenin pathway plays an important role in human CRC tumorigenesis [83]. Moreover, our very recent study identified that *FABP6* is the *Wnt* signaling pathway molecule which may indirectly interact with β-catenin [84]. Therefore, the correlations among *FABP6*, miR-320, and the *Wnt*/β-catenin pathway in CRC need to be further explored.

The tumor microenvironment plays an important role in tumor progression. Tumor-associated macrophages (TAMs) were reported to promote proliferation, invasion, metastasis, angiogenesis, and immunosuppression. High TAMs levels were significantly related to worse progression-free survival and a poorer response rate than low TAMs levels [85,86]. In addition to macrophages, another biomarker correlated with the cancer-associated systemic inflammatory response is increased circulating neutrophil counts. Neutrophils produce cytokines and chemokines, which are crucial in the development of cancer. Lymphocytes, on the other hand, can aid in the development of a cytotoxic immune response to cancer. Previous research has found that a reduction in serum lymphocyte counts has a detrimental impact on CRC patients’ prognoses [87,88,89]. The neutrophil–lymphocyte ratio (NLR) was found to be a significant surrogate measure for OS and cancer-specific survival (CSC) in a study of 12,118 patients, including 1413 CRC patients [90]. Numerous studies have shown that the NLR can be utilized as a major predictor of CRC patient survival, despite the fact that the characteristics of the patients recruited and the ideal NLR cutoff levels differed from study to study [91]. Another lymphocyte that helps coordinate the immune response to fight infection is CD4^+^ T lymphocytes, by stimulating other immune cells, such as macrophages, B lymphocytes (B cells), and CD8^+^ T lymphocytes (CD8 cells). CD4 is a glycoprotein that functions as a coreceptor for the T-cell receptor (TCR) [92]. The production of interleukin (IL)-1 and other cytokines by monocytes, macrophages, cancer cells, and fibroblasts was connected to the development of tumor-related immunosuppression, which might explain why IL-1 is associated with the development of COAD, and investigating this immune cell infiltration is critical [93]. The presence of heavy infiltration of tumor-infiltrating lymphocytes (TILs) in tumors, particularly CD4^+^ and CD8^+^ T cells, was linked to a favorable clinical prognosis for patients, as seen by prolonged progression-free survival (PFS) and OS [94,95,96,97].

We discovered a link between FABP6 expression and insulin-like growth factor (IGF) signaling in CRC. FABP6 expression was also linked to nearly all clinicopathologic variables and was associated with a worse outcome in CRC patients with a p53 mutation. FABP6 overexpression results in an increase in the abundance of interleukin (IL)-1 and IL-6. IL-1 is a powerful proinflammatory cytokine that plays a key protective role against infection and damage [98]. Inflammation, immunological responses, and hematopoiesis are all influenced by IL-6 [99]. As part of the inflammatory response, its signaling reduces IGF-binding protein 4 (*IBP4*) and *IBP2* protein production. These two genes belong to the *IBP* family of protein-coding genes. Inflammation and cytokine production by visceral adipose tissues might lead to alternations of inhibitor of nuclear factor-κBβ (IκBβ) and peroxisome proliferator-activated receptor (PPAR) signaling, which are potential therapeutic targets for insulin resistance and chemoprevention of CRC. Diseases associated with the IGF signaling pathway include CRC [100]. The IGF signaling pathway is a complicated and tightly controlled network that is essential for cell proliferation and survival [101]. According to some studies, high levels of IGF1 in circulation and an elevated IGF1/IGFBP3 ratio disrupt growth hormone (GH)/IGF1 balance, which might be a sign of cancer risk [102]. GRB10, PIK3R3, PIK3R1, and IRS1 were identified as differentiating transcripts in two investigations of expression patterns of genes encoding proteins of signaling cascades triggered by IGFs in CRC [103,104].

According to previous research, FABP4 was implicated in metabolic balance and inflammatory disorders [105,106,107]. FABP4, also known as adipocyte FABP (AFABP) or adipocyte P2 (aP2), is a small cytoplasmic FABP that is produced by macrophages, adipose tissues, and mature adipocytes. According to Zhang et al., serum FABP4 and FABP6 levels in Chinese patients can possibly be used as biomarkers for CRC diagnoses. FABP4 and FABP6 serum levels were substantially higher in CRC patients, and elevated FABP4 and FABP6 expression was linked to the development of CRC, according to their findings [108]. In our study, we found that overexpression of FABP4 and FABP6 were also related to a poor CRC OS prognosis.

Correlations between FABPs levels and clinical parameters, such as age, body-mass index (BMI), waist circumference, high-density lipoprotein (HDL) cholesterol, low-density lipoprotein (LDL) cholesterol, total cholesterol, systolic blood pressure, diastolic blood pressure, triglycerides, glucose, insulin, homeostasis model asssessmeent of insulin resistance (HOMA-R) (an indicator of insulin resistance), estimated glomerular filtration rate (eGFR), alanine aminotransferase (ALT), aspartate transaminase (AST), high sensitivity C-reactive protein (hsCRP), and brain natriuretic peptide (BNP), were previously investigated. According to a recent study, compared to normal conditions, increased FABP tissue concentrations and serum level concentrations make FABP serum levels unique and sensitive. However, that study only measured the concentrations and serum levels of FABP1–5 [109]. In this present study, *FABP6* mRNA expression was found to be significantly higher in the progression from normal tissues to adenomas and CRC. We hypothesize that *FABP6* overexpression in cancer cells is linked to early-stage carcinogenesis, but it is not required for late-stage cancer progression. Lastly, we assessed the biological role of overexpression of the *FABP6* gene in colorectal malignancies. Our results showed that *FABP6* could be a potential biomarker for CRC.

## 5. Conclusions

Our study showed that *FABP6* is overexpressed in CRC patients and is related to a poor prognosis in terms of overall survival prognosis. Through an enrichment pathway, we found that *FABP6* has an important role in early CRC as it was correlated with miRNAs linked to cell proliferation in the IGF pathway in CRC. In conclusion, *FABP6* could possibly serve as a potential prognostic biomarker and therapeutic target in CRC.

## Supplementary Materials

The following are available online at https://www.mdpi.com/article/10.3390/biomedicines9101460/s1: Figure S1: Correlations between differentially expressed fatty acid-binding protein (*FABP*) genes and immune cell infiltration in primary colorectal cancer (CRC) patients (A–F) (TIMER), Figure S2: Fatty acid-binding protein 1 (*FABP1*) differentially expressed gene pathways in colorectal cancer (CRC) developed by MetaCore. “Transcription_HIF-1 targets” were correlated with CRC development, Figure S3: Fatty acid-binding protein 2 (*FABP2*) differentially expressed gene pathways in colorectal cancer (CRC) developed by MetaCore. “PXR-mediated direct regulation of xenobiotic metabolizing enzymes human version” was correlated with CRC development, Figure S4: Fatty acid-binding protein 3 (*FABP3*) differentially expressed gene pathways in colorectal cancer (CRC) developed by MetaCore. “Immune response_Immunological synapse formation” was correlated with CRC development, Figure S5: Fatty acid-binding protein 4 (*FABP4*) differentially expressed gene pathways in colorectal cancer (CRC) developed by MetaCore. “Immune response_lectin-induced complement pathway” was correlated with CRC development, Figure S6: Fatty acid-binding protein 5 (*FABP5*) differentially expressed gene pathways in colorectal cancer (CRC) developed by MetaCore. “DNA damage_intra S-phase checkpoint” was correlated with CRC development, Figure S7: Fatty acid-binding protein 7 (*FABP7*) differentially expressed gene pathways in colorectal cancer (CRC) developed by MetaCore. “Immune response_IL-12 signaling pathway” was correlated with CRC development, Figure S8: Fatty acid-binding protein 9 (*FABP9*) differentially expressed gene pathways in colorectal cancer (CRC) developed by MetaCore. “Putative role of Tregs in COPD” was correlated with CRC development, Figure S9: Fatty acid-binding protein 12 (*FABP12*) differentially expressed gene pathways in colorectal cancer (CRC) developed by MetaCore. “Development_TGF-beta-dependent induction of EMT via MAPK” was correlated with CRC development, Table S1: Prognostic value of fatty acid-binding proteins (*FABPs*) in colorectal cancer (CRC) (PrognoScan database) (Cox *p* < 0.05), Table S2: *FABP1* differentially expressed genes pathway in CRC developed by MetaCore. Transcription_HIF-1 targets were correlated with CRC development, Table S3: *FABP2* differentially expressed genes pathway in CRC developed by MetaCore. PXR-mediated direct regulation of xenobiotic metabolizing enzymes human version was correlated with CRC development, Table S4: *FABP3* differentially expressed genes pathway in CRC developed by MetaCore. Immune response_immunological synapse formation was correlated with CRC development, Table S5: *FABP4* differentially expressed genes pathway in CRC developed by MetaCore. Immune response_lectin-induced complement pathway was correlated with CRC development, Table S6: *FABP5* differentially expressed genes pathway in CRC developed by MetaCore. DNA damage_intra S-phase checkpoint was correlated with CRC development, Table S7: *FABP6* differentially expressed genes pathway in CRC developed by MetaCore. Role of microRNAs in cell proliferation in colorectal cancer was correlated with CRC development, Table S8: *FABP7* differentially expressed genes pathway in CRC developed by MetaCore. Immune response_IL-12 signaling pathway was correlated with CRC development, Table S9: *FABP9* differentially expressed genes pathway in CRC developed by MetaCore. Putative role of Tregs in COPD was correlated with CRC development, Table S10: *FABP12* differentially expressed genes pathway in CRC developed by MetaCore. Development_TGF-beta-dependent induction of EMT via MAPK was correlated with CRC development.

## Abbreviations

FABPs, fatty-acid binding proteins; CRC, colorectal cancer; COAD, colon adenocarcinoma; READ, rectal adenocarcinoma; CCLE, Cancer Cell Line Encyclopedia; GEPIA, Gene Expression Profiling Interactive Analysis; STRING, Search Tool for the Retrieval of Interacting Genes/Proteins; TIMER, Tumor Immune Estimation Resource; OS, overall survival.
