# Supplementary material for: Expression Profiles and Prognostic Value of FABPs in Colorectal Adenocarcinomas
Source: Biomedicines. 2021 Oct 13;9(10):1460. doi: 10.3390/biomedicines9101460 (PMC8533171; doi:10.3390/biomedicines9101460)
Supplement: Supplementary file 1 [file biomedicines-09-01460-s001.zip › biomedicines-1365814-supplementary.pdf]

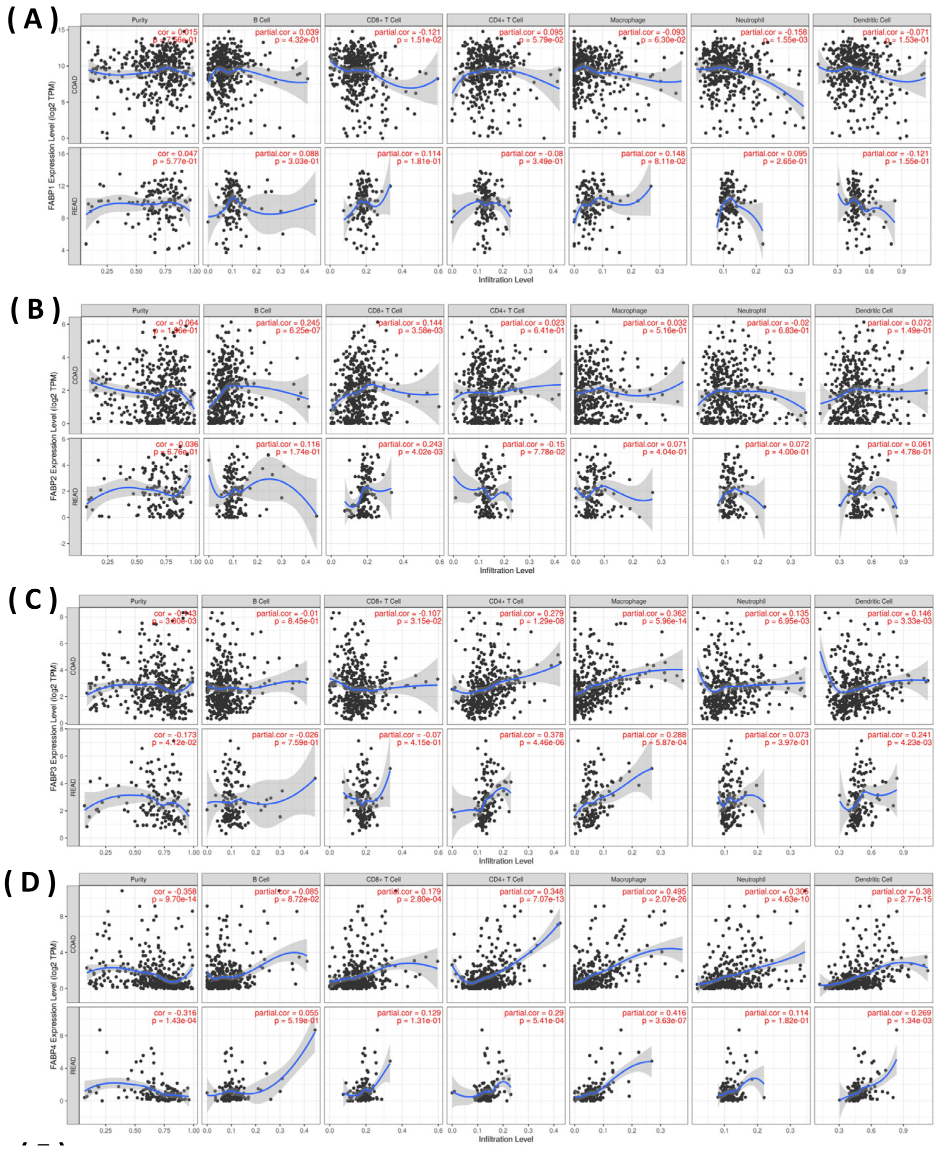
(A)
(B)
(C)
(D)
Purity
B Cell
CD8+ T Cell
CD4+ T Cell
Macrophage
Neutrophil
Dendritic Cell
COAD
READ
FABP1 Expression Level (log2 TPM)
FABP2 Expression Level (log2 TPM)
FABP3 Expression Level (log2 TPM)
FABP4 Expression Level (log2 TPM)
Infiltration Level

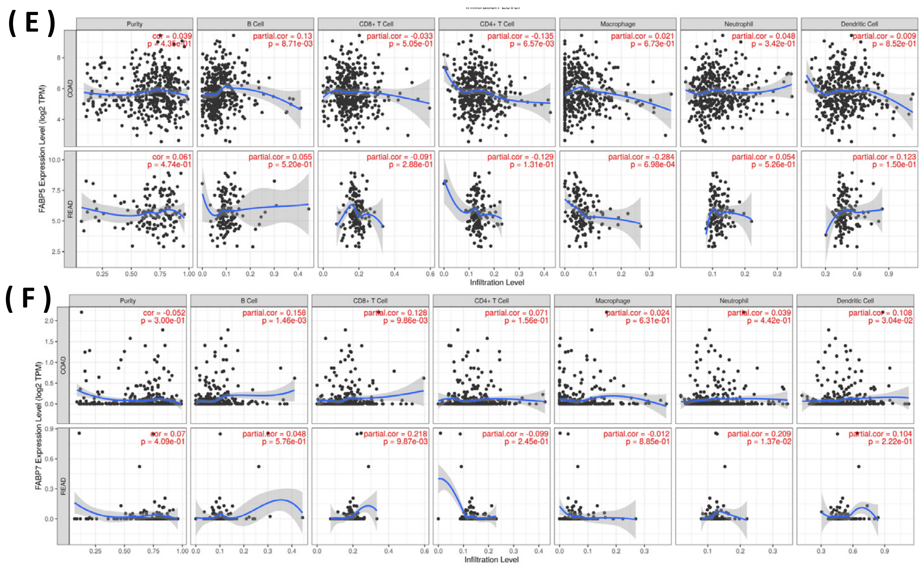

Figure S1. Correlations between differentially expressed fatty acid-binding protein (FABP) genes and immune cell infiltration in primary colorectal cancer (CRC) patients (A–F) (TIMER).

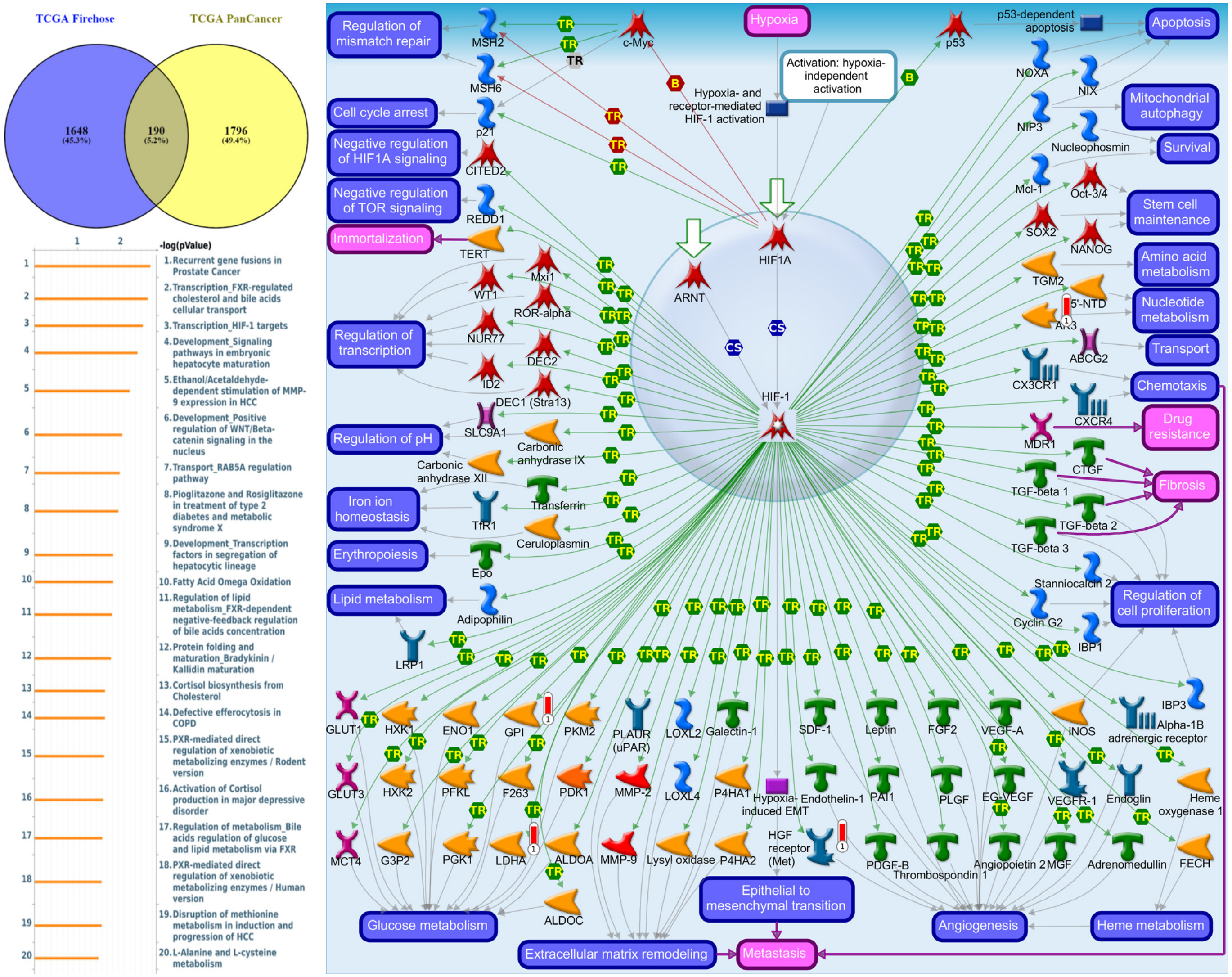

Figure S2. Fatty acid-binding protein 1 (FABP1) differentially expressed gene pathways in colorectal cancer (CRC) developed by MetaCore. "Transcription_HIF-1 targets" were correlated with CRC development.

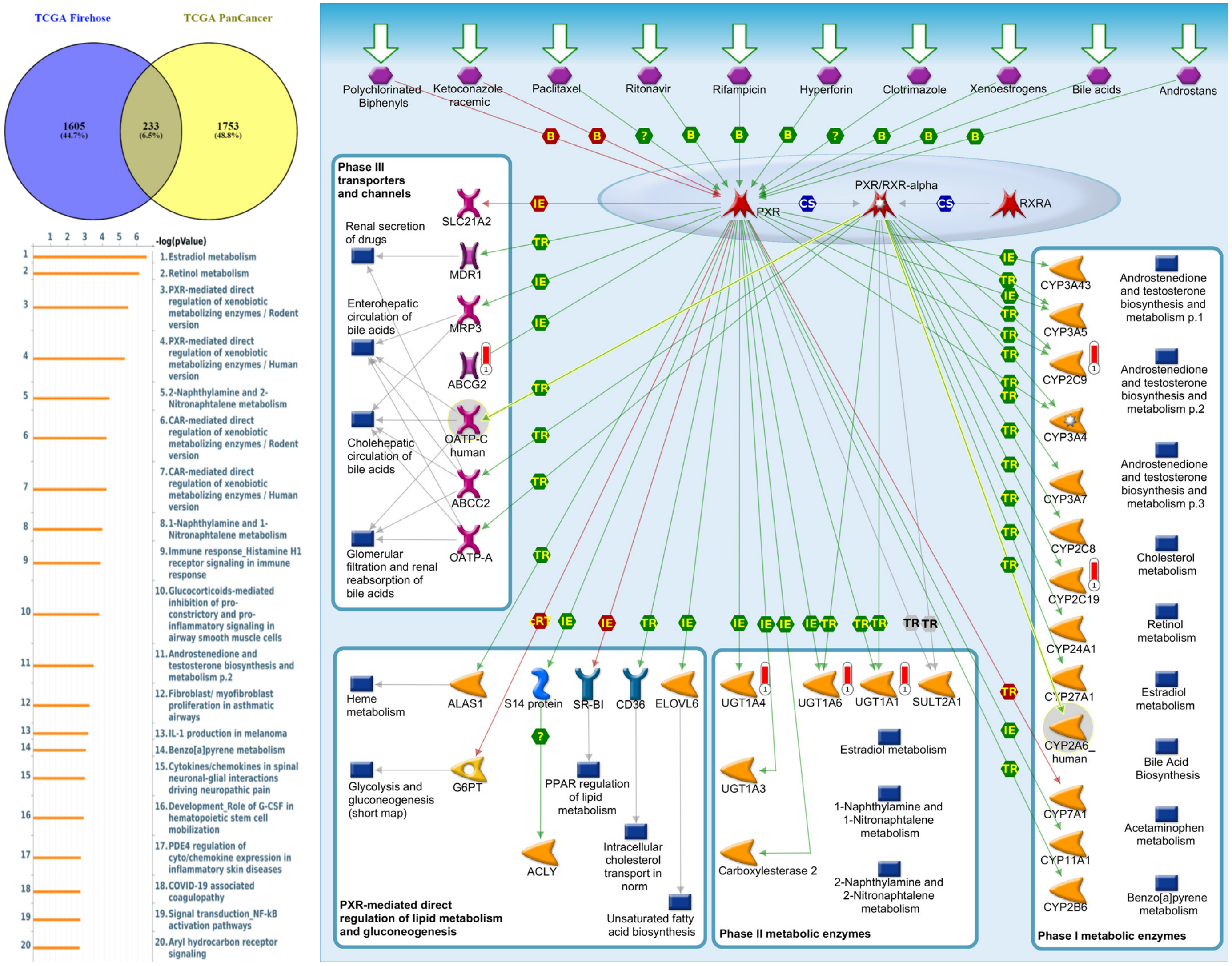

Figure S3. Fatty acid-binding protein 2 (FABP2) differentially expressed gene pathways in colorectal cancer (CRC) developed by MetaCore. "PXR-mediated direct regulation of xenobiotic metabolizing enzymes human version" was correlated with CRC development.

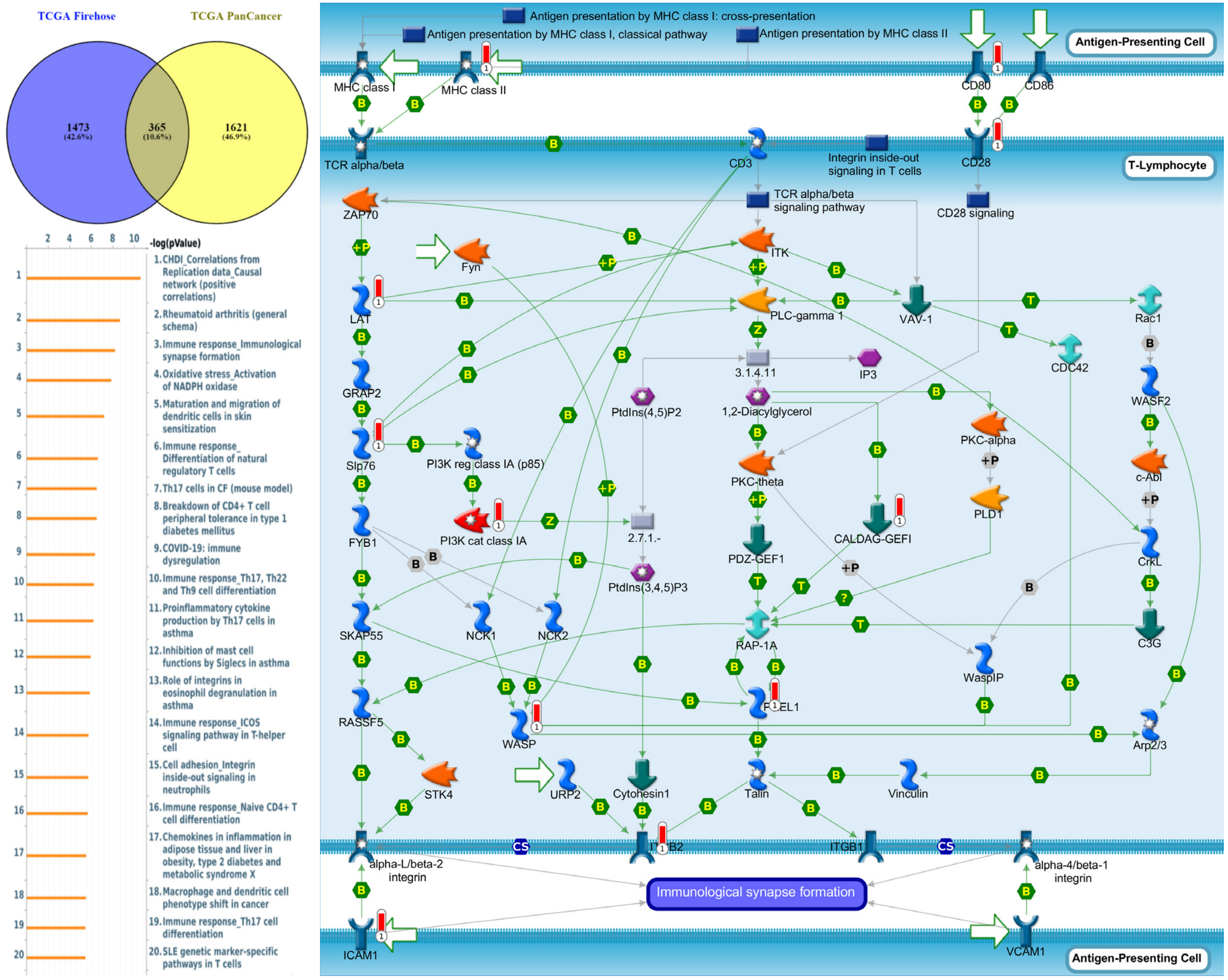

Figure S4. Fatty acid-binding protein 3 (FABP3) differentially expressed gene pathways in colorectal cancer (CRC) developed by MetaCore. "Immune response_Immunological synapse formation" was correlated with CRC development.

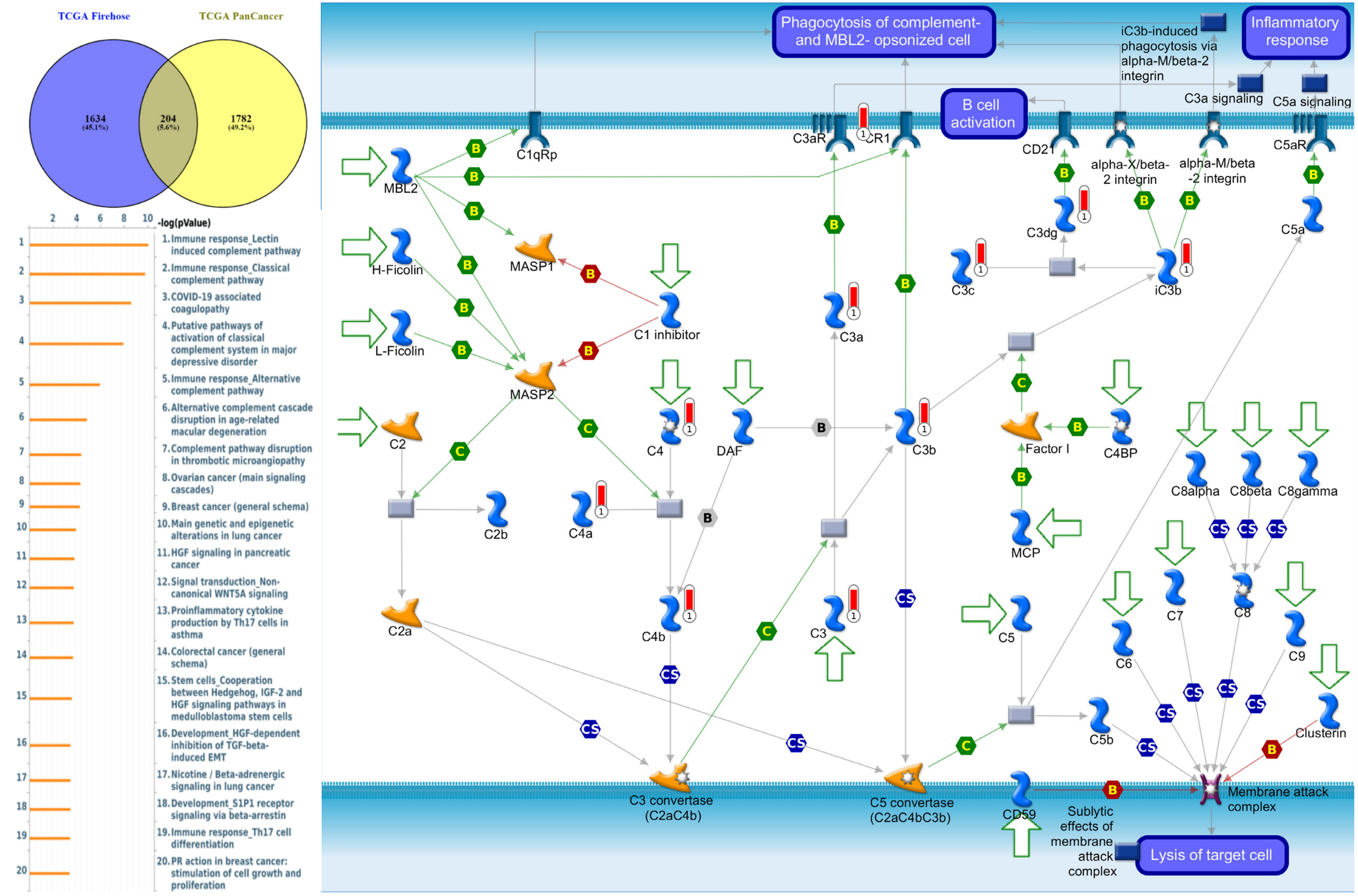

Figure S5. Fatty acid-binding protein 4 (FABP4) differentially expressed gene pathways in colorectal cancer (CRC) developed by MetaCore. "Immune response_Lectin-induced complement pathway" was correlated with CRC development.

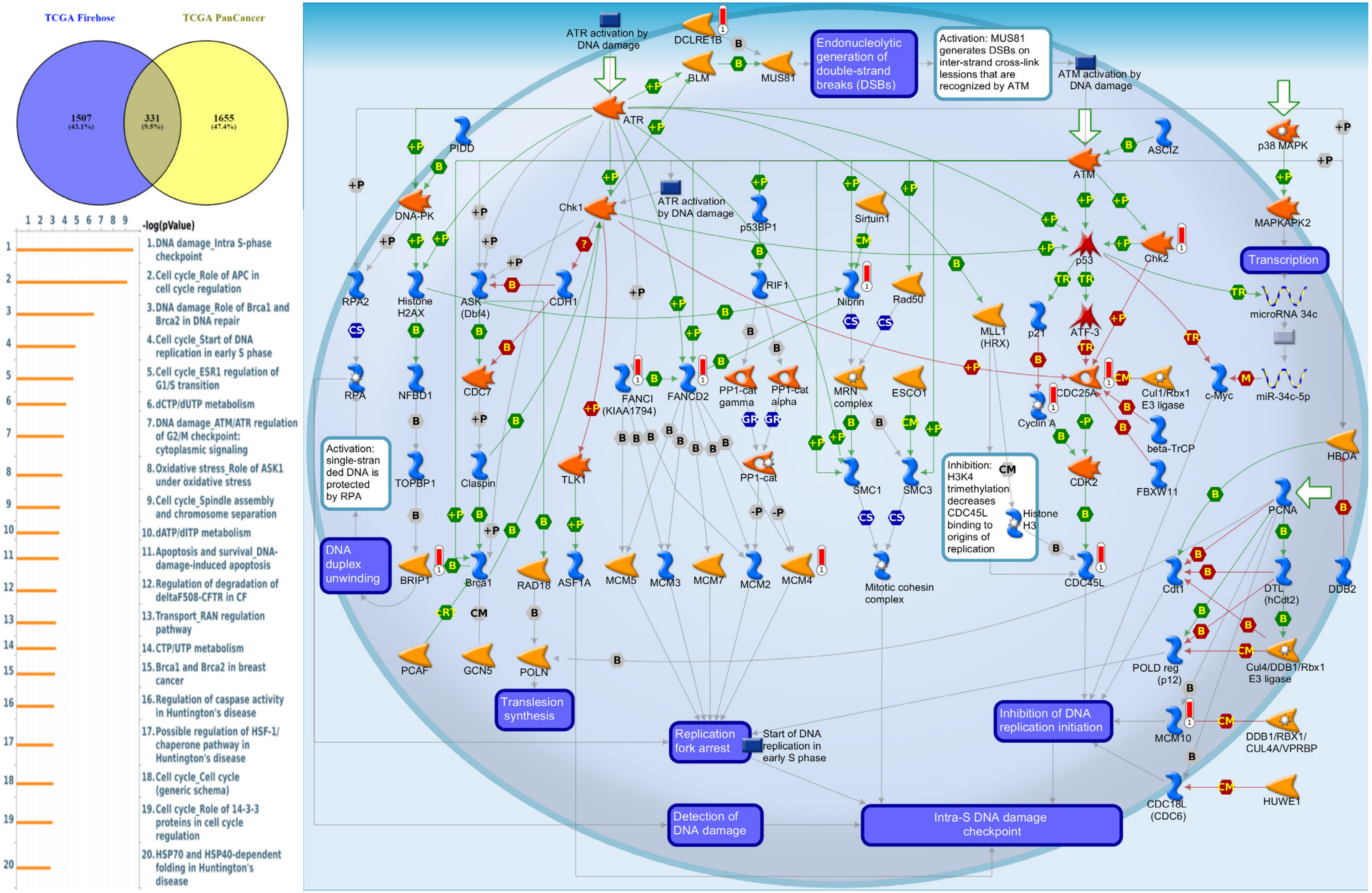

Figure S6. Fatty acid-binding protein 5 (FABP5) differentially expressed gene pathways in colorectal cancer (CRC) developed by MetaCore. "DNA damage_Intra S-phase checkpoint" was correlated with CRC development.

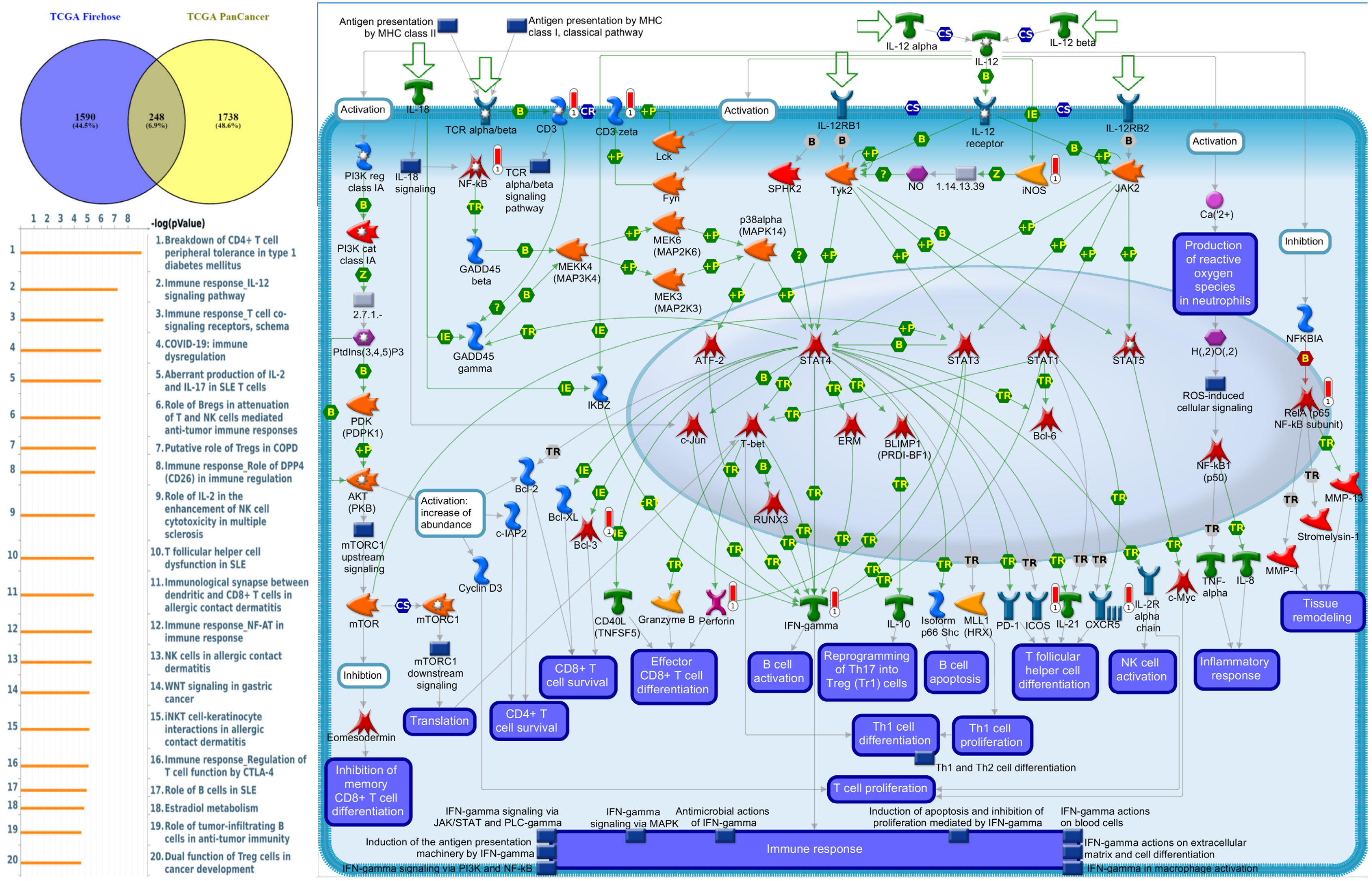

Figure S7. Fatty acid-binding protein 7 (FABP7) differentially expressed gene pathways in colorectal cancer (CRC) developed by MetaCore. "Immune response_IL-12 signaling pathway" was correlated with CRC development.

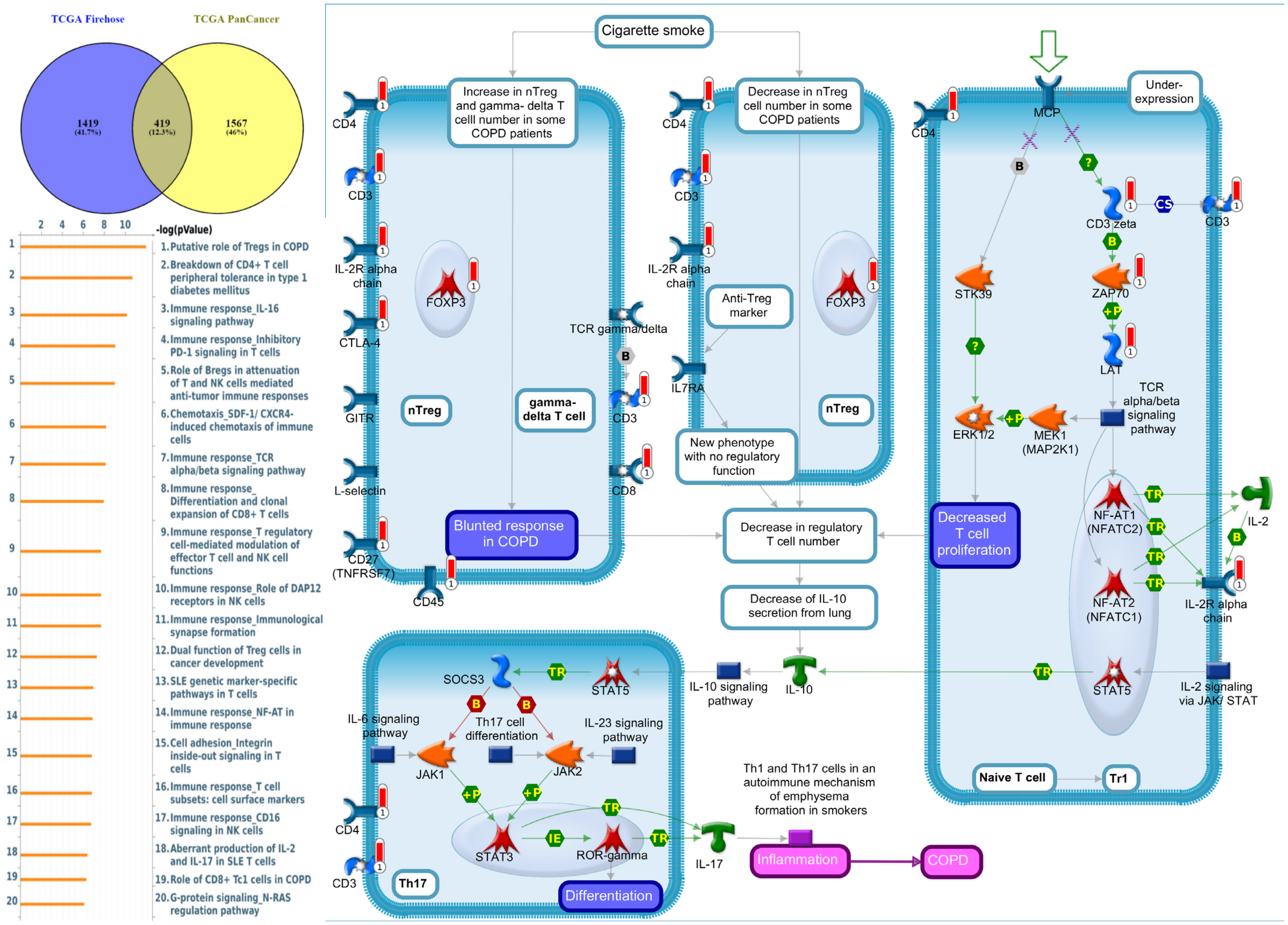

Figure S8. Fatty acid-binding protein 9 (FABP9) differentially expressed gene pathways in colorectal cancer (CRC) developed by MetaCore. "Putative role of Tregs in COPD" was correlated with CRC development.

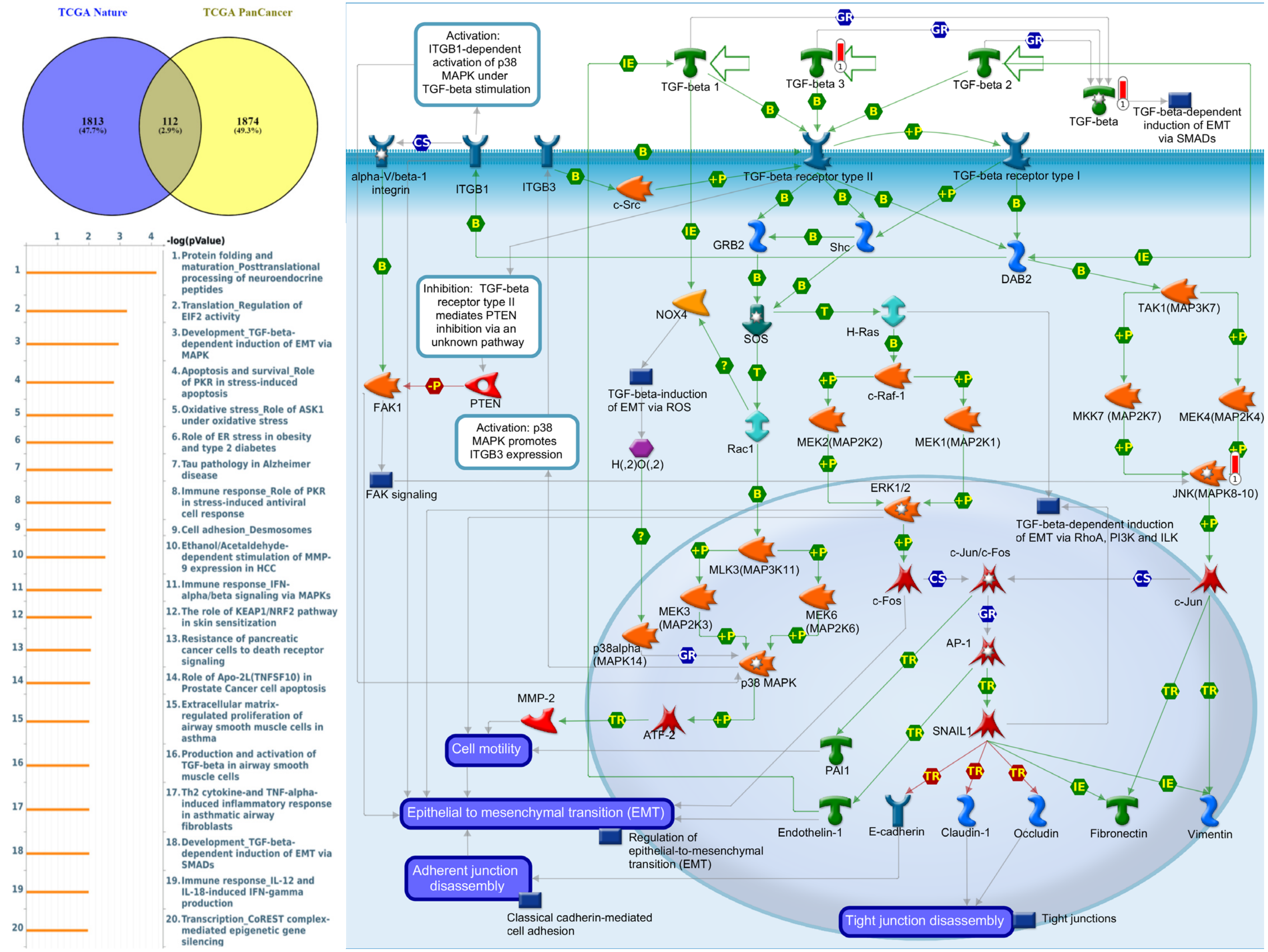

Figure S9. Fatty acid-binding protein 12 (FABP12) differentially expressed gene pathways in colorectal cancer (CRC) developed by MetaCore. "Development_TGF-beta-dependent induction of EMT via MAPK" was correlated with CRC development.

Table S1. Prognostic value of fatty acid-binding proteins (FABPs) in colorectal cancer (CRC) (PrognoScan database) (Cox p<0.05).

GENE	COX *P*-VALUE	ln(HR)	HR [95% CI]
**FABP1**	0.387885	0.26	1.30 [0.72 - 2.34]
**FABP2**	0.97584	-0.02	0.98 [0.27 - 3.58]
**FABP3**	0.734289	0.12	1.13 [0.56 - 2.27]
**FABP4**	0.027956	0.23	1.25 [1.02 - 1.54]
**FABP5**	0.444368	-0.29	0.75 [0.36 - 1.57]
**FABP6**	0.009886	0.41	1.51 [1.10 - 2.07]
**FABP7**	0.165921	-2.34	0.10 [0.00 - 2.63]

Table S2. FABP1 differentially expressed genes pathway in CRC developed by MetaCore. Transcription_HIF-1 targets was correlated with CRC development

#	Maps	pValue	Network Objects from Active Data
**1**	Recurrent gene fusions in Prostate Cancer	2.055E-03	4EHP, SLTM, TMPRSS2
**2**	Transcription_FXR-regulated cholesterol and bile acids cellular transport	2.366E-03	SR-BI, FXR, OSTalpha
**3**	Transcription_HIF-1 targets	3.094E-03	LDHA, AK3, GPI, HGF receptor (Met)
**4**	Development_Signaling pathways in embryonic hepatocyte maturation	4.120E-03	HNF1-alpha, FXR, HGF receptor (Met)
**5**	Ethanol/Acetaldehyde-dependent stimulation of MMP-9 expression in HCC	6.344E-03	ADH4, ADH7
**6**	Development_Positive regulation of WNT/Beta-catenin signaling in the nucleus	9.554E-03	TLE, NLK, NRARP
**7**	Transport_RAB5A regulation pathway	1.086E-02	ANKRD27(Varp), Syntaxin 4

**8**	Pioglitazone and Rosiglitazone in treatment of type 2 diabetes and metabolic syndrome X	1.172E-02	SR-BI, GPD1
**9**	Development_Transcription factors in segregation of hepatocytic lineage	1.543E-02	HNF1-alpha, HGF receptor (Met)
**10**	Fatty Acid Omega Oxidation	1.543E-02	ADH4, ADH7
**11**	Regulation of lipid metabolism_FXR-dependent negative-feedback regulation of bile acids concentration	1.643E-02	CYP2B6, FXR
**12**	Protein folding and maturation_Bradykinin / Kallidin maturation	1.745E-02	Carboxypeptidase M, Carboxypeptidase N (cat)
**13**	Cortisol biosynthesis from Cholesterol	2.413E-02	CYP11B2, CYP11B1
**14**	Defective efferocytosis in COPD	2.413E-02	TIM-1, TIM-4
**15**	PXR-mediated direct regulation of xenobiotic metabolizing enzymes / Rodent version	2.534E-02	CYP2B6, SR-BI
**16**	Activation of Cortisol production in major depressive disorder	2.656E-02	CYP11B2, CYP11B1
**17**	Regulation of metabolism_Bile acids regulation of glucose and lipid metabolism via FXR	2.781E-02	HNF1-alpha, FXR
**18**	PXR-mediated direct regulation of xenobiotic metabolizing enzymes / Human version	2.908E-02	CYP2B6, SR-BI
**19**	Disruption of methionine metabolism in induction and progression of HCC	2.908E-02	ADH4, ADH7
**20**	L-Alanine and L-cysteine metabolism	3.440E-02	LDHA, GPT2
**21**	Immune response_Induction of apoptosis and inhibition of proliferation mediated by IFN-gamma	3.578E-02	RIPK3, DAXX
**22**	Development_PIP3 signaling in cardiac myocytes	3.578E-02	PTP-1B, HGF receptor (Met)

**23**	Trichloroethylene metabolism	3.719E-02	CYP2B6, GGT1
**24**	Axonal degeneration in multiple sclerosis	3.861E-02	NCX2, Neurofascin
**25**	Immune response_Production and main functions of biologically active leukotrienes and Lipoxin A4	4.006E-02	ABCC6, GGT1
**26**	Cortisone biosynthesis and metabolism	4.152E-02	CYP11B2, CYP11B1
**27**	Alcohol metabolism predisposition of HCC development	4.301E-02	ADH4, ADH7
**28**	Immune response_T cell co-signaling receptors, schema	4.759E-02	TIM-1, TIM-4
**29**	Neurophysiological process_Constitutive and activity-dependent synaptic AMPA receptor delivery	5.396E-02	Dynamin-3, Syntaxin 4
**30**	Triacylglycerol metabolism p.1	5.559E-02	AK1BA, GPD1
**31**	Transcription_Effect of Folic acid on genome stability	5.559E-02	ADH4, HCP1
**32**	Development_WNT/Beta-catenin signaling in the nucleus	5.892E-02	TLE, Pitx2
**33**	Role of microRNAs in cell proliferation in colorectal cancer	7.107E-02	Rab-22A, HGF receptor (Met)
**34**	Retinol metabolism	7.287E-02	CYP2B6, CYP2C18
**35**	Development_Schema: Embryonal myogenesis	7.360E-02	HGF receptor (Met)
**36**	COVID-19: SARS-CoV-2 effects on infected tissues	7.563E-02	4EHP, SR-BI, TMPRSS2
**37**	Apoptosis and survival_FasL(TNFSF6)/ FasR(CD95)-induced cell death	7.651E-02	RIPK3, DAXX

**38**	Development_Role of nicotinamide in G-CSF-induced granulopoiesis	7.949E-02	ID1
**39**	Development_Positive regulation of WNT/Beta-catenin signaling in the cytoplasm	8.395E-02	HSP105, BIG2
**40**	Fructose metabolism	9.356E-02	AK1BA, GPI
**41**	Neurophysiological process_Kappa-type opioid receptor signaling in the central nervous system	9.552E-02	CACNA2D, SERT
**42**	Androgen biosynthetic pathways	9.552E-02	CYP11B2, CYP11B1
**43**	Cytoskeleton remodeling_Substance P mediated membrane blebbing	9.692E-02	Dynamin
**44**	DNA damage_Role of SUMO in p53 regulation	1.027E-01	DAXX
**45**	G-protein signaling_Rac3 regulation pathway	1.027E-01	HNF1-alpha
**46**	Development_Role of proteases in hematopoietic stem cell mobilization	1.084E-01	Carboxypeptidase M
**47**	Androgen signaling in HCC	1.084E-01	HGF receptor (Met)
**48**	Development_Negative regulation of WNT/Beta-catenin signaling in the nucleus	1.095E-01	TLE, NLK
**49**	Role of IL-8 in colorectal cancer	1.140E-01	HGF receptor (Met)
**50**	Immune response_ETV3 affect on CSF1-promoted macrophage differentiation	1.140E-01	ETV3

Table S3. FABP2 differentially expressed genes pathway in CRC developed by MetaCore. PXR-mediated direct regulation of xenobiotic metabolizing enzymes Human version was correlated with CRC development.

#	Maps	pValue	Network Objects from Active Data
**1**	Estradiol metabolism	2.732E-07	CYP2C19, CYP2C9, UGT1A10, UGT2B7, UGT1A1, UGT1A4, SULT1E1
**2**	Retinol metabolism	7.602E-07	CYP2C19, CYP2C9, UGT1A8, UGT2B7, DHA2, BCDO, AL1A1, CYP2C18
**3**	PXR-mediated direct regulation of xenobiotic metabolizing enzymes / Rodent version	3.298E-06	CYP2C19, CYP2C9, ABCG2, UGT1A1, UGT1A4, UGT1A6
**4**	PXR-mediated direct regulation of xenobiotic metabolizing enzymes / Human version	5.164E-06	CYP2C19, CYP2C9, ABCG2, UGT1A1, UGT1A4, UGT1A6
**5**	2-Naphthylamine and 2-Nitronaphtalene metabolism	4.209E-05	UGT1A8, GSTA1, UGT1A1, UGT1A4, UGT1A6, GSTA5
**6**	CAR-mediated direct regulation of xenobiotic metabolizing enzymes / Rodent version	6.375E-05	CYP2C19, CYP2C9, UGT1A1, AL1A1, UGT1A6
**7**	CAR-mediated direct regulation of xenobiotic metabolizing enzymes / Human version	6.375E-05	CYP2C19, CYP2C9, UGT1A1, AL1A1, UGT1A6
**8**	1-Naphthylamine and 1-Nitronaphtalene metabolism	1.134E-04	UGT1A8, GSTA1, UGT1A1, UGT1A4, UGT1A6
**9**	Immune response_Histamine H1 receptor signaling in immune response	1.400E-04	Tissue factor, RelA (p65 NF-kB subunit), Histamine H1 receptor, iNOS, cPLA2
**10**	Glucocorticoids-mediated inhibition of pro-constrictory and pro-inflammatory signaling in airway smooth muscle cells	1.710E-04	PLA2, RelA (p65 NF-kB subunit), PA24A, NF-kB, PDE4D
**11**	Androstenedione and testosterone biosynthesis and metabolism p.2	3.588E-04	UGT1A8, UGT1A10, UGT1A1, UGT1A4
**12**	Fibroblast/ myofibroblast proliferation in asthmatic airways	6.322E-04	Tissue factor, BDKRB1, Histamine H1 receptor, NF-kB

**13**	IL-1 production in melanoma	7.323E-04	MSK1, RelA (p65 NF-kB subunit), NF-kB
**14**	Benzo[a]pyrene metabolism	1.028E-03	CYP2C9, UGT1A10, CYP2C18, UGT1A6
**15**	Cytokines/chemokines in spinal neuronal-glial interactions driving neuropathic pain	1.164E-03	RelA (p65 NF-kB subunit), P2X4, ST2(L), NF-kB, iNOS, cPLA2
**16**	Development_Role of G-CSF in hematopoietic stem cell mobilization	1.388E-03	Cathepsin K, Carboxypeptidase M, GFI-1
**17**	PDE4 regulation of cyto/chemokine expression in inflammatory skin diseases	1.980E-03	MSK1, RelA (p65 NF-kB subunit), iNOS, PDE4
**18**	COVID-19 associated coagulopathy	2.131E-03	Tissue factor, DAF, NF-kB, cPLA2
**19**	Signal transduction_NF-kB activation pathways	2.131E-03	BAFF-R, RelA (p65 NF-kB subunit), NF-kB, TRAF3
**20**	Aryl hydrocarbon receptor signaling	2.456E-03	ABCG2, GSTA1, UGT1A1, UGT1A6
**21**	Immune response_HMGB1/RAGE signaling pathway	2.456E-03	Tissue factor, NF-kB, iNOS, Chromogranin A
**22**	Apoptosis and survival_NO synthesis and signaling	2.814E-03	NF-kB, iNOS, CaMK IV, NF-kB p65/p65
**23**	Development_Generation of pancreatic beta-cells from induced pluripotent stem cells	2.913E-03	KLF4, NeuroD1, GLUT2
**24**	Th2-cytokines and IFN-mediated regulation of airway smooth muscle contraction	2.913E-03	RelA (p65 NF-kB subunit), Histamine H1 receptor, IL13RA1
**25**	Apoptosis and survival_Anti-apoptotic TNFs/NF-kB/IAP pathway	2.913E-03	RelA (p65 NF-kB subunit), NF-kB, TRAF3
**26**	Immune response_IL-15 signaling via MAPK and PI3K cascades	3.006E-03	IL-15, RelA (p65 NF-kB subunit), NF-kB, iNOS
**27**	Immune response_IL-33 signaling pathway	3.416E-03	MSK1, RelA (p65 NF-kB subunit), ST2(L), NF-kB

**28**	Cigarette smoke-mediated regulation of NRF2-antioxidant pathway in airway epithelial cells	3.583E-03	ME1, GSTA1, UGT1A4
**29**	Retinoic acid maintains mucocilliary differentiation and mucins expression in normal and asthmatic airway epithelium	3.950E-03	Mucin 4, GCNT3, Mucin 2
**30**	Signal transduction_MIF signaling pathway	4.099E-03	MSK1, RelA (p65 NF-kB subunit), NF-kB, cPLA2
**31**	Arachidonic acid metabolites production in alveolar macrophages in asthma	4.339E-03	NF-kB, PLA2(hGIIA), cPLA2
**32**	Linoleic acid metabolism	4.339E-03	CYP2C19, PA24A, PLA2(hGIIA)
**33**	Acetaminophen metabolism	4.603E-03	UGT1A10, ABCG2, UGT1A1, UGT1A6
**34**	O-glycan biosynthesis	4.603E-03	B3Gn-T6, GCNT3, B3GT1, B3GT5
**35**	Vascular endothelial cell damage in SLE	4.603E-03	Tissue factor, RelA (p65 NF-kB subunit), NF-kB, iNOS
**36**	TNF-alpha and IL-1 beta-mediated regulation of contraction and secretion of inflammatory factors in normal and asthmatic airway smooth muscle	5.146E-03	PLA2, RelA (p65 NF-kB subunit), PA24A, NF-kB
**37**	Canonical WNT signaling pathway in colorectal cancer	5.434E-03	Galectin-3, iNOS, IAP, Mucin 2
**38**	Inflammatory mechanisms of pancreatic cancerogenesis	5.731E-03	RelA (p65 NF-kB subunit), Mucin 4, NF-kB, iNOS
**39**	B-regulatory cells and tumor cells intercellular interaction	5.731E-03	RelA (p65 NF-kB subunit), ST2(L), NF-kB, NF-kB p65/p65
**40**	Oxidative stress_ROS-induced cellular signaling	6.051E-03	PAX5, Bak, RelA (p65 NF-kB subunit), NF-kB, iNOS
**41**	Interleukins-induced inflammatory response in asthmatic airway fibroblasts	6.124E-03	MSK1, RelA (p65 NF-kB subunit), NF-kB

**42**	Estrone metabolism	6.124E-03	CYP2C19, CYP2C9, SULT1E1
**43**	NF-kB pathway in multiple myeloma	6.124E-03	BAFF-R, NF-kB, TRAF3
**44**	Keratan sulfate metabolism p.1	6.124E-03	B3GN7, B3GN6, CHST6
**45**	Th2 cytokine- and TNF-alpha-induced inflammatory response in asthmatic airway fibroblasts	6.124E-03	RelA (p65 NF-kB subunit), NF-kB, IL13RA1
**46**	Th2-cytokine-induced airway epithelium mucous metaplasia in COPD	6.690E-03	NF-kB, iNOS, NF-kB p65/p65, IL13RA1
**47**	Apoptosis and survival_APRIL and BAFF signaling	8.290E-03	BAFF-R, RelA (p65 NF-kB subunit), TRAF3
**48**	Androstenedione and testosterone biosynthesis and metabolism p.3	8.893E-03	UGT1A10, UGT2B7, UGT2B4
**49**	B cell signaling in hematological malignancies	9.325E-03	BAFF-R, NF-kB, CD20, TRAF3
**50**	Apoptosis and survival_Anti-apoptotic TNFs/NF-kB/Bcl-2 pathway	1.017E-02	RelA (p65 NF-kB subunit), NF-kB, TRAF3

Table S4. FABP3 differentially expressed genes pathway in CRC developed by MetaCore. Immune response_Immunological synapse formation was correlated with CRC development.

#	Maps	pValue	Network Objects from Active Data
**1**	CHDI_Correlations from Replication data_Causal network (positive correlations)	2.635E-11	PI3K cat class IA, CD83, ICAM1, CD28, HSP70, IP3R1, MEF2, LAT, Slp76, IP3 receptor, PI3K reg class IB (p101), NR2A, CD80, MHC class II, NR2
**2**	Rheumatoid arthritis (general schema)	2.392E-09	ICAM1, MHC class II beta chain, CD28, FOXP3, HLA-DRB, CD4, HLA-DRB1, TGF-beta, CSF1, CD80, MHC class II

**3**	Immune response_Immunological synapse formation	7.031E-09	PI3K cat class IA, ITGB2, ICAM1, CD28, LAT, Slp76, CALDAG-GEFI, WASP, CD80, PREL1, MHC class II
**4**	Oxidative stress_Activation of NADPH oxidase	1.535E-08	PI3K cat class IA, Tiam1, PKC-beta2, PI3K reg (p87-gamma), gp91-phox, p47-phox, IP3 receptor, PKC, p67-phox, PI3K reg class IB (p101), cPKC (conventional)
**5**	Maturation and migration of dendritic cells in skin sensitization	7.221E-08	MHC class II alpha chain, CD83, ICAM1, MHC class II beta chain, HLA-DRB, HLA-DRB1, HLA-DRB3, CD80, MHC class II
**6**	Immune response_Differentiation of natural regulatory T cells	2.807E-07	JAK3, CD28, FOXP3, TGF-beta 1, CD4, TGF-beta receptor type I, CD80, MHC class II
**7**	Th17 cells in CF (mouse model)	3.696E-07	JAK3, ICAM1, CD28, FOXP3, TGF-beta 1, CD4, TGF-beta receptor type I, CD80, MHC class II
**8**	Breakdown of CD4+ T cell peripheral tolerance in type 1 diabetes mellitus	3.696E-07	JAK3, CD28, Bcl-6, FOXP3, LAT, CD4, HLA-DRB1, CD80, MHC class II
**9**	COVID-19: immune dysregulation	5.377E-07	HLA-DPB1, CCR5, HLA-DPA1, TLR7, FOXP3, GATA-3, GATA Group, TGF-beta 1, CD4, HLA-DRB1, CSF1, MHC class II
**10**	Immune response_Th17, Th22 and Th9 cell differentiation	6.858E-07	PU.1, CD28, GATA-3, TGF-beta 1, CD4, TGF-beta receptor type I, CD80, MHC class II
**11**	Proinflammatory cytokine production by Th17 cells in asthma	7.448E-07	JAK3, C5aR, CD28, C3aR, GATA-3, TGF-beta 1, CD4, CD80, MHC class II
**12**	Inhibition of mast cell functions by Siglecs in asthma	1.375E-06	Siglec-8, Siglec-10, LAT, CD33, CD22, Slp76, IP3 receptor
**13**	Role of integrins in eosinophil degranulation in asthma	1.644E-06	PI3K cat class IA, Plastin, Hck, ICAM1, Substance P receptor, PKC-beta2, PKC, FGR, CSF2RB
**14**	Immune response_ICOS signaling pathway in T-helper cell	2.206E-06	PI3K cat class IA, c-Maf, CD28, Tiam1, Bcl-6, FOXP3, IP3 receptor, CD4, MHC class II
**15**	Cell adhesion_Integrin inside-out signaling in neutrophils	2.329E-06	ITGB2, Hck, ICAM1, Slp76, CALDAG-GEFI, IP3 receptor, PI3K reg class IB (p101), FGR, PREL1, P-selectin
**16**	Immune response_Naive CD4+ T cell differentiation	2.585E-06	c-Maf, PU.1, CD28, GATA-3, TGF-beta 1, CD4, CD80, MHC class II

**17**	Chemokines in inflammation in adipose tissue and liver in obesity, type 2 diabetes and metabolic syndrome X	3.615E-06	ICAM1, CCR5, CMKLR1, CD68, CD163, MANR, MHC class II, P-selectin
**18**	Macrophage and dendritic cell phenotype shift in cancer	3.840E-06	ESR1 (nuclear), M-CSF receptor, TLR7, ILT4, ILT3, TGF-beta 1, TGF-beta receptor type I, CSF1, CD80, MHC class II, TLR9
**19**	Immune response_Th17 cell differentiation	4.180E-06	JAK3, CD28, TGF-beta 1, CD4, TGF-beta receptor type I, CD80, MHC class II
**20**	SLE genetic marker-specific pathways in T cells	4.236E-06	CalDAG-GEFIII, ESR1 (nuclear), Ikaros, MHC class II beta chain, IP3R1, LAT, HLA-DRB, Slp76, HLA-DRB1, HLA-DRB3, MHC class II
**21**	Immune response_IFN-gamma in macrophages activation	4.972E-06	c-Maf, PU.1, C1qb, Selenoprotein P, gp91-phox, C1qc, p67-phox, TLR9
**22**	B-regulatory cells and tumor cells intercellular interaction	5.665E-06	c-Maf, JAK3, CD28, TLR7, Bcl-6, TGF-beta 1, TGF-beta receptor type I, CD80, TLR9
**23**	Development_Glucocorticoid receptor signaling	6.750E-06	GCR Alpha, GCR, HSP70, GCR Beta, TGF-beta receptor type I, Oct-2
**24**	Immune response_Inhibitory PD-1 signaling in T cells	7.805E-06	PI3K cat class IA, ICAM1, CD28, FOXP3, GATA-3, CD4, CD80, MHC class II
**25**	Complement pathway disruption in thrombotic microangiopathy	8.946E-06	C5aR, C3aR, C1q, Factor H, PKC, cPKC (conventional), P-selectin
**26**	Th17 cells in CF	9.009E-06	JAK3, ICAM1, CD28, TGF-beta 1, CD4, TGF-beta receptor type I, CD80, MHC class II
**27**	T follicular helper cell dysfunction in SLE	9.729E-06	PI3K cat class IA, c-Maf, CD84, JAK3, CD28, TLR7, Bcl-6, CD4, CD80, MHC class II
**28**	Th1 and Th17 cells in an autoimmune mechanism of emphysema formation in smokers	1.367E-05	Alpha 1-antitrypsin, CD28, CCR5, CD4, CD80, MHC class II
**29**	Bone metastases in Prostate Cancer	1.696E-05	PTHR1, IGF-1, Frizzled, RUNX2, Kallikrein 3 (PSA), WNT

**30**	Immune response _CCR3 signaling in eosinophils	1.804E-05	Hck, Tiam1, gp91-phox, p47-phox, IP3 receptor, p67-phox, PI3K reg class IB (p101), WASP, FGR
**31**	Immune response_M-CSF-receptor signaling pathway	2.225E-05	PI3K cat class IA, PU.1, M-CSF receptor, Hck, GAB3, IP3 receptor, PKC, WASP, CSF1
**32**	Immune response_Antigen presentation by MHC class I: cross-presentation	2.273E-05	Syntaxin 4, TLR7, HSP70, C1q, gp91-phox, p47-phox, IP-30, p67-phox, MANR, TLR9
**33**	CHDI_Correlations from Replication data_Cytoskeleton and adhesion module	3.240E-05	ITGB2, ICAM1, Slp76, CALDAG-GEFI, IP3 receptor, PI3K reg class IB (p101), WASP, MHC class II
**34**	Oxidative stress in adipocyte dysfunction in type 2 diabetes and metabolic syndrome X	3.240E-05	PI3K cat class IA, PU.1, PLA2, APOE, Heme oxygenase 1, gp91-phox, p47-phox, p67-phox
**35**	Dysregulation of germinal center response in SLE	3.633E-05	ESR1 (nuclear), JAK3, ICAM1, TLR7, Bcl-6, PI3K cat class IA (p110-delta), Oct-2, CD21
**36**	Renal tubulointerstitial injury in Lupus Nephritis	3.633E-05	M-CSF receptor, ICAM1, CCR5, CMKLR1, TGF-beta 1, CD4, CSF1, MHC class II
**37**	Oxidative stress_Role of IL-8 signaling pathway in respiratory burst	3.688E-05	PKC-beta2, PKC-beta1, gp91-phox, p47-phox, IP3 receptor, p67-phox, PI3K reg class IB (p101)
**38**	Origin of Langerhans cells in the steady-state and under inflammatory conditions	5.459E-05	M-CSF receptor, TGF-beta 1, TGF-beta receptor type I, CSF1, P-selectin
**39**	Immune response_NF-AT in immune response	5.517E-05	PI3K cat class IA, CD28, LAT, Slp76, IP3 receptor, CD80, MHC class II
**40**	Role of IL-17-producing T cells in allergic contact dermatitis	6.865E-05	ICAM1, CD28, CD4, CD80, MHC class II
**41**	Inhibition of neutrophil migration by proresolving lipid mediators in COPD	6.925E-05	ITGB2, C5aR, ICAM1, CCR5, IP3 receptor, PKC, PI3K reg class IB (p101), cPKC (conventional)
**42**	Fibroblast/ myofibroblast proliferation in asthmatic airways	7.282E-05	PI3K cat class IA, EDNRB, IGF-1, TGF-beta 1, TGF-beta receptor type I, PKC
**43**	Signal transduction_Calcium-mediated signaling	7.661E-05	Tiam1, PPA5, MEF2, PKC-beta, p47-phox, IP3 receptor, PKC, cPKC (conventional)

**44**	Immune response_IL-3 signaling via JAK/STAT, p38, JNK and NF-kB	8.205E-05	PI3K cat class IA, PU.1, JAK3, ICAM1, Bcl-6, C3aR, CSF2RB, MHC class II, P-selectin
**45**	CHDI_Correlations from Discovery data_Causal network	8.461E-05	PI3K cat class IA, Substance P receptor, MEF2, Frizzled, IP3 receptor, TGF-beta receptor type I, PI3K reg class IB (p101), WNT
**46**	T cell generation in COPD	1.050E-04	ICAM1, CD28, CD4, CD80, MHC class II
**47**	Immune response_TCR alpha/beta signaling pathway	1.139E-04	CD28, LAT, P2X7, Slp76, IP3 receptor, CD4, WASP, CD80, MHC class II
**48**	Immune response_Th1 and Th2 cell differentiation	1.145E-04	c-Maf, CD28, GATA-3, CD4, CD80, MHC class II
**49**	Role of Bregs in attenuation of T and NK cells mediated anti-tumor immune responses	1.320E-04	Adenosine A2a receptor, CD28, FOXP3, TGF-beta 1, TGF-beta receptor type I, CD80
**50**	Breast cancer (general schema)	1.320E-04	PTHR1, ESR1 (nuclear), PTCH1, ESR1 (membrane), TGF-beta receptor type I, TGF-beta

Table S5. FABP4 differentially expressed genes pathway in CRC developed by MetaCore. Immune response_Lectin induced complement pathway was correlated with CRC development.

#	Maps	pValue	Network Objects from Active Data
1	Immune response_Lectin induced complement pathway	9.417E-11	C3b, C4, C3aR, iC3b, C3dg, C3a, C3, C4a, C3c, C4b
2	Immune response_Classical complement pathway	1.740E-10	C3b, C4, C3aR, iC3b, C3dg, C3a, C3, C4a, C3c, C4b
3	COVID-19 associated coagulopathy	2.762E-09	C3b, Coagulation factor VIII, C4, IL-6, C3aR, C3a, C3, PSGL-1, C4b
4	Putative pathways of activation of classical complement system in major depressive disorder	1.290E-08	C3b, C4A protein, C4, IL-6, C3, C4B protein, C4b

**5**	Immune response_Alternative complement pathway	1.346E-06	C3b, C3aR, iC3b, C3dg, C3a, C3, C3c
**6**	Alternative complement cascade disruption in age-related macular degeneration	1.716E-05	C3b, C3aR, iC3b, C3a, C3
**7**	Complement pathway disruption in thrombotic microangiopathy	5.430E-05	C3b, C3aR, C3a, C3, PSGL-1
**8**	Ovarian cancer (main signaling cascades)	6.377E-05	HGF, G-protein alpha-i family, IL-6, SOS, HGF receptor (Met), Tcf(Lef)
**9**	Breast cancer (general schema)	6.948E-05	IL-6, DHH, TGF-beta receptor type I, PR (membrane), PR (nuclear)
**10**	Main genetic and epigenetic alterations in lung cancer	1.496E-04	MSP, HGF, IBP, HGF receptor (Met), IBP3
**11**	HGF signaling in pancreatic cancer	2.052E-04	HGFA, HGF, SOS, HGF receptor (Met)
**12**	Signal transduction_Non-canonical WNT5A signaling	2.333E-04	ROR2, G-protein alpha-i family, Lef-1, NF-AT2(NFATC1), NF-AT, G-protein alpha-o
**13**	Proinflammatory cytokine production by Th17 cells in asthma	2.403E-04	ROR-alpha, IL-6, C3aR, C3a, C3
**14**	Colorectal cancer (general schema)	2.702E-04	HGF, Ephrin-B receptors, IL-6, HGF receptor (Met)
**15**	Stem cells_Cooperation between Hedgehog, IGF-2 and HGF signaling pathways in medulloblastoma stem cells	3.488E-04	HGF, Lef-1, HGF receptor (Met), Tcf(Lef)
**16**	Development_HGF-dependent inhibition of TGF-beta-induced EMT	4.424E-04	HGF, SOS, HGF receptor (Met), TGF-beta receptor type I
**17**	Nicotine / Beta-adrenergic signaling in lung cancer	4.424E-04	G-protein alpha-i family, SOS, Beta-2 adrenergic receptor, Beta-adrenergic receptor
**18**	Development_S1P1 receptor signaling via beta-arrestin	4.424E-04	G-protein alpha-i family, G-protein alpha-i2, SOS, G-protein alpha-o
**19**	Immune response_Th17 cell differentiation	4.955E-04	ROR-alpha, NF-AT2(NFATC1), IL-6, TGF-beta receptor type I

**20**	PR action in breast cancer: stimulation of cell growth and proliferation	5.528E-04	SOS, Cyclin A, PR (membrane), PR (nuclear)
**21**	MAPK-independent proliferation of normal and asthmatic smooth muscle cells	5.815E-04	G-protein alpha-i family, EDNRB, CysLT1 receptor, TGF-beta receptor type I, IBP3
**22**	Eosinophil chemotaxis in asthma	5.815E-04	G-protein alpha-i family, C3aR, Secretoneurin, CysLT1 receptor, C3a
**23**	Neurophysiological process_Constitutive and regulated NMDA receptor trafficking	6.246E-04	PACAP, G-protein alpha-i family, Chapsyn-110, Neuroligin 1, SNAP-25
**24**	Immune response_Th17, Th22 and Th9 cell differentiation	7.532E-04	ROR-alpha, NF-AT2(NFATC1), IL-6, TGF-beta receptor type I
**25**	Reproduction_Progesterone-mediated oocyte maturation	8.300E-04	G-protein alpha-i family, SOS, PR (membrane), CPEB1
**26**	Development_Muscle progenitor cell migration in hypaxial myogenesis	9.122E-04	HGF, G-protein alpha-i family, SOS, HGF receptor (Met)
**27**	Stromal-epithelial interaction in Prostate Cancer	1.000E-03	HGF, IL-6, HGF receptor (Met), TGF-beta receptor type I
**28**	Inhibition of WNT5A-dependent non-canonical pathway in colorectal cancer	1.005E-03	ROR-alpha, ROR2, Lef-1
**29**	Regulation and signaling of HGF receptor (Met) and MSP receptor (RON) in lung cancer	1.062E-03	MSP, HGF, IL-6, SOS, HGF receptor (Met)
**30**	Development_ACM2 and ACM4 activation of ERK	1.094E-03	G-protein alpha-i family, G-protein alpha-i2, SOS, G-protein alpha-o
**31**	Signal transduction_BMP signaling via ALK-1 and ALK-2 receptors	1.359E-03	BMP6, ALK-2, ALK-1
**32**	Inflammatory response in ischemia-reperfusion injury during myocardial infarction	1.359E-03	IL-6, C3a, PSGL-1
**33**	Development_HGF signaling pathway	1.530E-03	HGFA, HGF, SOS, HGF receptor (Met)
**34**	EGF- and HGF-dependent stimulation of metastasis in gastric cancer	1.561E-03	HGF, SOS, HGF receptor (Met)

**35**	Immune response_C3a signaling	1.655E-03	G-protein alpha-i family, IL-6, C3aR, C3a
**36**	Muscle contraction_GPCRs in the regulation of smooth muscle tone	1.886E-03	TRPC4, G-protein alpha-i family, Beta-2 adrenergic receptor, CysLT1 receptor, MyHC
**37**	Inflammatory factors-induced expression of mucins in normal and asthmatic epithelium	2.074E-03	IL-6, SOS, C3aR, C3a
**38**	IGF signaling in HCC	2.229E-03	HGF, SOS, HGF receptor (Met), IBP3
**39**	Influence of bone marrow cell environment on progression of multiple myeloma	2.229E-03	CD23, HGF, IL-6, HGF receptor (Met)
**40**	EGFR signaling pathway in lung cancer	2.391E-03	HGF, IL-6, SOS, HGF receptor (Met)
**41**	Neurophysiological process_GABA-B receptor signaling at postsynaptic sides of synapses	2.555E-03	G-protein alpha-i family, Kir3.4, G-protein alpha-o
**42**	Regulation of AKT(PKB)/ GSK3 beta cascade in bipolar disorder	2.561E-03	G-protein alpha-i family, G-protein alpha-i2, GFRalpha1, SOS
**43**	Growth factors in regulation of oligodendrocyte precursor cells proliferation in multiple sclerosis	2.561E-03	PLP1, HGF, SOS, HGF receptor (Met)
**44**	Desensitization of Beta-2 adrenergic receptor signaling and reduction of relaxation	2.852E-03	G-protein alpha-i family, SOS, Beta-2 adrenergic receptor
**45**	MAPK-mediated proliferation of normal and asthmatic smooth muscle cells	2.926E-03	G-protein alpha-i family, EDNRB, SOS, TGF-beta receptor type I
**46**	Airway smooth muscle contraction in asthma	2.926E-03	G-protein alpha-i family, Beta-2 adrenergic receptor, CysLT1 receptor, MyHC
**47**	Neurophysiological process_GABA-B receptor signaling in presynaptic nerve terminals	3.170E-03	G-protein alpha-i family, G-protein alpha-o, SNAP-25
**48**	Impaired inhibition of Th17 cell differentiation by IFN-beta in multiple sclerosis	3.170E-03	ROR-alpha, IL-6, TGF-beta receptor type I
**49**	Development_Hedgehog signaling	3.249E-03	G-protein alpha-i family, DHH, BOC, CARD8, EVC2

**50**	Macrophage-induced immunosuppression in the tumor microenvironment	3.720E-03	MSP, ILT2, NF-AT2(NFATC1), IL-6, ENP1

Table S6. FABP5 differentially expressed genes pathway in CRC developed by MetaCore. DNA damage_Intra S-phase checkpoint was correlated with CRC development.

#	Maps	pValue	Network Objects from Active Data
**1**	DNA damage_Intra S-phase checkpoint	2.309E-10	Nibrin, CDC25A, MCM10, FANCD2, DCLRE1B, BRIP1, Chk2, MCM4, Cyclin A, CDC45L, FANCI (KIAA1794)
**2**	Cell cycle_Role of APC in cell cycle regulation	7.311E-10	Kid, CDC25A, CDC20, MAD2a, Emi1, ORC1L, Cyclin A, PLK1
**3**	DNA damage_Role of Brca1 and Brca2 in DNA repair	4.529E-07	Nibrin, FANCD2, BRIP1, Chk2, Rad51, DNA polymerase beta
**4**	Cell cycle_Start of DNA replication in early S phase	1.554E-05	MCM10, ORC6L, MCM4, ORC1L, CDC45L
**5**	Cell cycle_ESR1 regulation of G1/S transition	2.446E-05	Cyclin A2, CDC25A, NCOA3 (pCIP/SRC3), CRM1, Cyclin A
**6**	dCTP/dUTP metabolism	9.839E-05	POLG reg, NDPK B, POLE3 (YBL1), DNA polymerase beta, AK2, AK3
**7**	DNA damage_ATM/ATR regulation of G2/M checkpoint: cytoplasmic signaling	1.556E-04	CDC25A, JAB1, Chk2, PLK1, 14-3-3
**8**	Oxidative stress_Role of ASK1 under oxidative stress	2.045E-04	MT-TRX, HPK38, 14-3-3 zeta/delta, Thioredoxin, 14-3-3
**9**	Cell cycle_Spindle assembly and chromosome separation	3.209E-04	Kid, CDC20, TPX2, MAD2a
**10**	dATP/dITP metabolism	3.671E-04	POLG reg, NDPK B, POLE3 (YBL1), DNA polymerase beta, AK2, AK3

**11**	Apoptosis and survival_DNA-damage-induced apoptosis	4.162E-04	Nibrin, FANCD2, Chk2
**12**	Regulation of degradation of deltaF508-CFTR in CF	6.156E-04	HSP90, Derlin1, Sti1, HSP70
**13**	Transport_RAN regulation pathway	7.300E-04	RanBP1, NUP54, CRM1
**14**	CTP/UTP metabolism	7.327E-04	RRP40, RRP4, CTP synthase, NDPK B, AK2, AK3
**15**	Brca1 and Brca2 in breast cancer	8.606E-04	Nibrin, BRIP1, Rad51
**16**	Regulation of caspase activity in Huntington's disease	1.005E-03	HSP90, HSP70, Caspase-3
**17**	Possible regulation of HSF-1/ chaperone pathway in Huntington's disease	1.164E-03	HSP90, HSP70, PLK1
**18**	Cell cycle_Cell cycle (generic schema)	1.164E-03	CDC25A, E2F5, Cyclin A
**19**	Cell cycle_Role of 14-3-3 proteins in cell cycle regulation	1.338E-03	CDC25A, 14-3-3 zeta/delta, Chk2
**20**	HSP70 and HSP40-dependent folding in Huntington's disease	1.955E-03	HSP90, Sti1, HSP70
**21**	Immune response_IFN-alpha/beta signaling via PI3K and NF-kB pathways	2.577E-03	NMI, CDC25A, I-TAC, eIF4E, Cyclin A
**22**	Mitogenic action of Estradiol / ESR1 (nuclear) in breast cancer	2.723E-03	CDC25A, NCOA3 (pCIP/SRC3), Cyclin E2
**23**	Development_Role of CNTF and LIF in regulation of oligodendrocyte development	2.723E-03	Caspase-3, IMPA1, 14-3-3
**24**	Cell cycle_Role of SCF complex in cell cycle regulation	3.015E-03	CDC25A, Emi1, PLK1
**25**	Transcription_Ligand-dependent activation of the ESR1/SP pathway	3.326E-03	CDC25A, NCOA3 (pCIP/SRC3), Cyclin E2

**26**	Role of CNTF and LIF in regulation of oligodendrocyte development in multiple sclerosis	3.326E-03	Caspase-3, IMPA1, 14-3-3
**27**	dGTP metabolism	3.919E-03	POLG reg, NDPK B, POLE3 (YBL1), DNA polymerase beta
**28**	TTP metabolism	4.871E-03	POLG reg, NDPK B, POLE3 (YBL1), DNA polymerase beta
**29**	Transcription_Role of the non-genomic action of Retinoic acid and phosphorylation of Retinoic acid receptors in the initiation of transcription	5.169E-03	MSK1, Cyclin H, NCOA3 (pCIP/SRC3)
**30**	DNA damage_Nucleotide excision repair	5.400E-03	NAP2, Histone H2A, HMG14, TFIIS
**31**	Cell cycle_The metaphase checkpoint	5.598E-03	CDC20, MAD2a, PLK1
**32**	Development_Growth factors in regulation of oligodendrocyte precursor cell survival	6.048E-03	14-3-3 zeta/delta, Caspase-3, Lyn
**33**	Cell cycle_Regulation of G1/S transition (part 1)	6.518E-03	CDC25A, Chk2, Cyclin A
**34**	ATP/ITP metabolism	8.057E-03	RRP40, RRP4, NDPK B, AK2, AK3
**35**	Cell cycle_Nucleocytoplasmic transport of CDK/Cyclins	8.470E-03	CRM1, Cyclin A
**36**	Growth factors in regulation of oligodendrocyte precursor cells survival in multiple sclerosis	8.616E-03	14-3-3 zeta/delta, Caspase-3, Lyn
**37**	Apoptosis and survival_TNF-alpha-induced Caspase-8 signaling	9.195E-03	HSP90, FAN, Caspase-3
**38**	DNA damage_ATM/ATR regulation of G1/S checkpoint	9.796E-03	CDC25A, Chk2, Cyclin A
**39**	DNA damage_ATM/ATR regulation of G2/M checkpoint: nuclear signaling	1.042E-02	Chk2, Cyclin A, PLK1

**40**	Mechanisms of deltaF508 CFTR activation by S-nitrosoglutathione	1.173E-02	HSP90, Sti1, HSP70
**41**	LRRK2 in neuronal apoptosis in Parkinson's disease	1.241E-02	Thioredoxin, Caspase-3
**42**	Development_GM-CSF signaling	1.388E-02	14-3-3 zeta/delta, Caspase-3, Lyn
**43**	Immune response_ETV3 affect on CSF1-promoted macrophage differentiation	1.540E-02	DDX20, MSK1/2 (RPS6KA5/4)
**44**	Immune response_Induction of the antigen presentation machinery by IFN-gamma	1.623E-02	PRMT5, PSME2, IFNGR1
**45**	Regulation of degradation of wtCFTR	1.700E-02	HSP90, Derlin1
**46**	Role of XBP1 protein in multiple myeloma	1.700E-02	PSMA6, PSMA5
**47**	Immune response_Antigen presentation by MHC class I, classical pathway	1.706E-02	PSME2, HSP70, PSMB2
**48**	Transcription_Epigenetic regulation of gene expression	1.970E-02	Histone H2A, PRMT5, SUV39H2
**49**	COVID-19: SARS-CoV-2 effects on infected tissues	2.024E-02	Thioredoxin, I-TAC, 4EHP, SRP72, MSK1/2 (RPS6KA5/4)
**50**	Inhibition of RUNX3 signaling in gastric cancer	2.040E-02	JAB1, Caspase-3

Table S7. FABP6 differentially expressed genes pathway in CRC developed by MetaCore. Role of microRNAs in cell proliferation in colorectal cancer was correlated with CRC development.

#	Maps	pValue	Network Objects from Active Data
1	Ubiquinone metabolism	2.456E-04	NDUFS5, NDUFB10, NDUFS4, NDUFB3, DAP13
2	L-Lysine metabolism	7.072E-04	PIPOX, CRYM, SLC38A4, AGPHD1, AL7A1
3	Role of microRNAs in cell proliferation in colorectal cancer	1.872E-03	CDK4, DNMT3A, RhoB, c-Jun
4	Prolactin signaling in Prostate Cancer	1.980E-03	AP-1, c-Jun, AATM
5	Immune response_Oncostatin M signaling via MAPK	2.759E-03	PPAR-gamma, AP-1, c-Jun
6	Autocrine Somatotropin signaling in breast cancer	3.209E-03	C/EBP zeta, DNMT3A, PDF
7	Glycine and L-Serine metabolism	3.446E-03	PIPOX, GCST, SLC38A4, SLC38A1, AL7A1
8	Epigenetic alterations in ovarian cancer	3.513E-03	CDK4, DNMT3A, FGFR3, AL1A1
9	Dual role of p53 in transcription deregulation in Huntington's Disease	3.606E-03	CBF2, MAP1LC3A
10	Regulation of Tissue factor signaling in cancer	4.240E-03	AP-1, c-Jun/c-Jun, c-Jun
11	Apoptosis and survival_TNFR1 signaling pathway	4.240E-03	Caspase-6, c-Jun, Smac/Diablo
12	Development_Ligand-dependent activation of the ESR1/AP-1 pathway	5.652E-03	AP-1, c-Jun

**13**	Proteases and EGFR-activated mucin production in airway epithelium in COPD	6.480E-03	PPAR-gamma, AP-1, c-Jun
**14**	Development_FGF10 in development of subcutaneous white adipose tissue in embryogenesis	7.249E-03	PPAR-gamma, c-Jun
**15**	Neurophysiological process_Dynein-dynactin motor complex in axonal transport in neurons	8.023E-03	DYI2, Dynein 1, cytoplasmic, intermediate chains, PRNP
**16**	Signal transduction_Angiotensin II signaling via Beta-arrestin	9.311E-03	DGK, Casein kinase II, beta chain (Phosvitin), MRLC
**17**	Cytoskeleton remodeling_Regulation of actin cytoskeleton organization by the kinase effectors of Rho GTPases	9.765E-03	RhoA-related, MRLC, RhoB
**18**	L-Methionine metabolism	1.023E-02	DNMT3A, SLC38A4, SLC38A1
**19**	Triacylglycerol metabolism p.1	1.071E-02	AL1B1, AL7A1, ALDH2
**20**	Immune response_IL-4-induced regulators of cell growth, survival, differentiation and metabolism	1.171E-02	PPAR-gamma, AP-1, CDK4
**21**	Metabolism of L-cysteine, D-cysteine and L-cystine	1.289E-02	SLC38A4, GSHB, SLC38A1, AATM
**22**	Tyrosine metabolism p.1 (dopamine)	1.623E-02	AL1B1, AL1A1, ALDH2
**23**	CREB1-dependent transcription deregulation in Huntington's Disease	1.657E-02	PPAR-gamma, COX VIa-1
**24**	Immune response_IL-6 signaling pathway via JAK/STAT	1.685E-02	AP-1, CDK4, c-Jun
**25**	Chemotaxis_Lysophosphatidic acid signaling via GPCRs	1.688E-02	AP-1, c-Jun, Rho GTPase, TRIP6
**26**	Role of IFN-beta in the improvement of blood-brain barrier integrity in multiple sclerosis	1.781E-02	AP-1, c-Jun
**27**	Histamine metabolism	1.781E-02	AL1B1, ALDH2

**28**	Immune response_IL-2 signaling via ERK, PI3K, and PLC-gamma	1.814E-02	CDK4, c-Myb, c-Jun
**29**	L-Phenylalanine metabolism	1.881E-02	AL1A1, ALDH2, AATM
**30**	Glucocorticoid- and LABA-mediated inhibition of pro-inflammatory signaling in airway fibroblasts/myofibroblasts	1.908E-02	AP-1, c-Jun
**31**	Cell cycle_Regulation of G1/S transition (part 2)	1.908E-02	CDK4, c-Jun
**32**	Catecholamine metabolism	1.948E-02	AL1B1, AL1A1, ALDH2
**33**	Proteolysis_Putative SUMO-1 pathway	2.040E-02	c-Myb, c-Jun
**34**	Signal transduction_Angiotensin II/ AGTR1 signaling via RhoA and JNK	2.160E-02	AP-1, MRLC, c-Jun
**35**	Role of histone modificators in progression of multiple myeloma	2.175E-02	CDK4, Dynein 1, cytoplasmic, intermediate chains
**36**	Leucine, isoleucine and valine metabolism.p.2	2.233E-02	HADHA, MCCC2, ALDH2
**37**	Role of inhibition of WNT signaling in the progression of lung cancer	2.313E-02	PPAR-gamma, c-Jun
**38**	Putative glucocorticoid- and LABA-mediated inhibition of pro-fibrotic signaling in airway fibroblasts/myofibroblasts	2.313E-02	AP-1, c-Jun
**39**	Inhibition of TGF-beta signaling in lung cancer	2.313E-02	CDK4, c-Jun
**40**	Influence of low doses of Arsenite on glucose uptake in adipocytes	2.313E-02	PPAR-gamma, KIF3B
**41**	Epithelial cell apoptosis in COPD	2.313E-02	PLAGL2, Prohibitin
**42**	Putative pathways for stimulation of fat cell differentiation by Bisphenol A	2.456E-02	Esrrg, PPAR-gamma

**43**	IL-2 as a growth factor for T cells in multiple sclerosis	2.601E-02	CDK4, c-Myb
**44**	Apoptosis and survival_Granzyme B signaling	2.601E-02	Caspase-6, Smac/Diablo
**45**	Peroxisomal branched chain fatty acid oxidation	2.619E-02	HADHA, HACL1, PAHX
**46**	Development_Histone acetylation in embryonic stem cells	2.750E-02	DNMT3A, NODAL
**47**	Production and activation of TGF-beta in airway smooth muscle cells	2.903E-02	AP-1, c-Jun
**48**	Cell cycle_ESR1 regulation of G1/S transition	2.903E-02	CDK4, c-Jun
**49**	HBV signaling via protein kinases leading to HCC	3.058E-02	AP-1, c-Jun
**50**	GTP-XTP metabolism	3.126E-02	POLR2C, NDPK 8, NDPK A

Table S8. FABP7 differentially expressed genes pathway in CRC developed by MetaCore. Immune response_IL-12 signaling pathway was correlated with CRC development.

#	Maps	pValue	Network Objects from Active Data
1	Breakdown of CD4+ T cell peripheral tolerance in type 1 diabetes mellitus	1.002E-09	CXCR5, ICOS, PTPN22, ZAP70, CD3, LAT, IFN-gamma, NF-kB, PD-L1, CTLA-4
2	Immune response_IL-12 signaling pathway	6.510E-08	CD3 zeta, CXCR5, RelA (p65 NF-kB subunit), ICOS, CD3, Perforin, IFN-gamma, NF-kB, iNOS, Bcl-3
3	Immune response_T cell co-signaling receptors, schema	7.872E-07	CD2, CD27(TNFRSF7), LAG3, ICOS, DPP4, CD244, PD-L1, CTLA-4
4	COVID-19: immune dysregulation	1.152E-06	UNC93B, RelA (p65 NF-kB subunit), CD3, Perforin, IFN-gamma, NF-kB, iNOS, CCR7, NKG2A, Granzyme A
5	Aberrant production of IL-2 and IL-17 in SLE T cells	1.196E-06	CD3 zeta, RelA (p65 NF-kB subunit), CD229, ZAP70, CD3, LAT, CD3 epsilon, SAP
6	Role of Bregs in attenuation of T and NK cells mediated anti-tumor immune responses	1.282E-06	CD3 zeta, LAG3, CD3, FasL(TNFSF6), IFN-gamma, PD-L1, CTLA-4
7	Putative role of Tregs in COPD	2.865E-06	CD3 zeta, CD27(TNFRSF7), ZAP70, CD3, LAT, CTLA-4
8	Immune response_Role of DPP4 (CD26) in immune regulation	3.346E-06	CD3 zeta, ZAP70, CD3, DPP4, FasL(TNFSF6), IFN-gamma, NF-kB
9	Role of IL-2 in the enhancement of NK cell cytotoxicity in multiple sclerosis	3.512E-06	FasL(TNFSF6), Perforin, IFN-gamma, NF-kB, Bcl-3, Granzyme A
10	T follicular helper cell dysfunction in SLE	4.010E-06	CXCR5, BAFF-R, ICOS, IRF4, SAP, TRIM, IFN-gamma, NF-kB, CTLA-4
11	Immunological synapse between dendritic and CD8+ T cells in allergic contact dermatitis	4.274E-06	CD2, CD27(TNFRSF7), ICOS, NF-kB, PD-L1, CTLA-4
12	Immune response_NF-AT in immune response	5.880E-06	CD3 zeta, ITK, ZAP70, CD3, LAT, TRIM, NF-kB

**13**	NK cells in allergic contact dermatitis	6.199E-06	FasL(TNFSF6), Perforin, IFN-gamma, NF-kB, NKp46, NKG2A
**14**	WNT signaling in gastric cancer	8.775E-06	WNT10B, WNT2B, IFN-gamma, WNT9A, WNT1, WNT
**15**	iNKT cell-keratinocyte interactions in allergic contact dermatitis	8.775E-06	ITK, CD161, CD3, CD3 epsilon, FasL(TNFSF6), IFN-gamma
**16**	Immune response_Regulation of T cell function by CTLA-4	1.035E-05	CD3 zeta, ZAP70, CD3, LAT, NF-kB, CTLA-4
**17**	Role of B cells in SLE	1.408E-05	Bak, BAFF-R, ICOS, IFN-gamma, NF-kB, RelB (NF-kB subunit), CD22
**18**	Estradiol metabolism	2.207E-05	CYP2C19, UGT1A10, UGT2B7, UGT1A1, UGT1A4, SULT1E1
**19**	Role of tumor-infiltrating B cells in anti-tumor immunity	3.603E-05	CXCR5, CD27(TNFRSF7), IRF4, FasL(TNFSF6), Perforin, IFN-gamma, NF-kB, NY-ESO-1
**20**	Dual function of Treg cells in cancer development	3.764E-05	ICOS, FasL(TNFSF6), Perforin, IFN-gamma, CTLA-4, WNT
**21**	Immune response_CD16 signaling in NK cells	4.448E-05	CD3 zeta, ZAP70, LAT, FasL(TNFSF6), IFN-gamma, Cytohesin1, NKG2A
**22**	Immune response_NF-AT signaling and leukocyte interactions	5.431E-05	ITK, ZAP70, CD3, LAT, FasL(TNFSF6), NF-kB
**23**	B cell signaling in hematological malignancies	9.038E-05	Cyclin D2, BAFF-R, CD27(TNFRSF7), ZAP70, NF-kB, CD22, TRAF3
**24**	Immune response_CD28 signaling	1.051E-04	ITK, ZAP70, CD3, LAT, NF-kB, PIP5KI
**25**	2-Naphthylamine and 2-Nitronaphtalene metabolism	1.715E-04	UGT1A8, GSTA1, UGT1A1, UGT1A4, UGT1A6, GSTA5
**26**	Multiple sclerosis (general schema)	1.748E-04	FasL(TNFSF6), IFN-gamma, WNT9A, CTLA-4
**27**	Immune response_Differentiation and clonal expansion of CD8+ T cells	1.864E-04	CD27(TNFRSF7), FasL(TNFSF6), Perforin, IFN-gamma, NF-kB

**28**	Role of iNKT and B cells in T cell recruitment in allergic contact dermatitis	1.864E-04	ITK, CXCR5, CD3, IFN-gamma, NF-kB
**29**	B-regulatory cells and tumor cells intercellular interaction	3.158E-04	RelA (p65 NF-kB subunit), FasL(TNFSF6), NF-kB, EBI3, PD-L1, NF-kB p65/p65
**30**	Role of CD8+ Tc1 cells in COPD	3.326E-04	CXCR6, IL-15, Perforin, IFN-gamma, NF-kB
**31**	Generation of cytotoxic CD8+ T cells in COPD	3.539E-04	Perforin, IFN-gamma, NF-kB, CTLA-4
**32**	Apo-2L(TNFSF10)-induced apoptosis in melanoma	3.700E-04	DR4(TNFRSF10A), Bak, RelA (p65 NF-kB subunit), IFN-gamma, NF-kB
**33**	1-Naphthylamine and 1-Nitronaphtalene metabolism	3.700E-04	UGT1A8, GSTA1, UGT1A1, UGT1A4, UGT1A6
**34**	Immune response_Role of HMGB1 in dendritic cell maturation and migration	4.138E-04	IL-15, IFN-gamma, NF-kB, CCR7
**35**	Immune response_Induction of apoptosis and inhibition of proliferation mediated by IFN-gamma	4.544E-04	KLF4, IL-15, WARS, FasL(TNFSF6), IFN-gamma
**36**	SLE genetic marker-specific pathways in T cells	4.900E-04	CD3 zeta, PTPN22, ZAP70, CD3, LAT, IFN-gamma, NF-kB
**37**	Immune response_IFN-gamma actions on blood cells	5.547E-04	IL-15, IFN-gamma, K12, PD-L1
**38**	G protein-coupled receptors signaling in lung cancer	6.245E-04	RelA (p65 NF-kB subunit), Galpha(q)-specific peptide GPCRs, Galpha(i)-specific peptide GPCRs, SSTR2, SSTR5, SSTR3
**39**	Signal transduction_NF-kB activation pathways	6.656E-04	BAFF-R, RelA (p65 NF-kB subunit), NF-kB, RelB (NF-kB subunit), TRAF3
**40**	Role of activation of WNT signaling in the progression of lung cancer	6.697E-04	WNT10B, WNT2B, iNOS, WNT9A, WNT1, WNT
**41**	Cell adhesion_Integrin inside-out signaling in T cells	7.283E-04	CD3 zeta, ZAP70, CD3, PIP5KI, CCR7
**42**	Immune response_T cell subsets: cell surface markers	7.283E-04	CD161, LAG3, CD3, CCR7, CTLA-4

**43**	Role of IL-23/ T17 pathogenic axis in psoriasis	7.953E-04	IL-15, Beta-defensin 3, IRF4, NF-kB, IL-1F5
**44**	Immune response_CCL2 signaling	8.667E-04	CCBP2 (CCR9), IFN-gamma, NF-kB, iNOS, p47-phox
**45**	Androstenedione and testosterone biosynthesis and metabolism p.2	9.332E-04	UGT1A8, UGT1A10, UGT1A1, UGT1A4
**46**	The role of KEAP1/NRF2 pathway in skin sensitization	9.332E-04	Heme oxygenase 1, IFN-gamma, NF-kB, CCR7
**47**	Upregulation of IL-8 expression in colorectal cancer	9.332E-04	RelA (p65 NF-kB subunit), IFN-gamma, WNT1, WNT
**48**	Immune response_Role of DAP12 receptors in NK cells	9.429E-04	KLRC3, ZAP70, LAT, NKG2C, NKG2A
**49**	Immune response_Immunological synapse formation	9.429E-04	ITK, ZAP70, CD3, LAT, Cytohesin1
**50**	Immune response_IFN-gamma signaling via PI3K and NF-kB	1.024E-03	RelA (p65 NF-kB subunit), IFN-gamma, NF-kB, iNOS, PD-L1

Table S9. FABP9 differentially expressed genes pathway in CRC developed by MetaCore. Putative role of Tregs in COPD was correlated with CRC development.

#	Maps	pValue	Network Objects from Active Data
**1**	Putative role of Tregs in COPD	1.077E-12	CD8, CD27(TNFRSF7), ZAP70, CD3, FOXP3, LAT, CTLA-4, IL-2R alpha chain, CD3 zeta, CD45, CD4
**2**	Breakdown of CD4+ T cell peripheral tolerance in type 1 diabetes mellitus	2.241E-11	STAT4, ZAP70, CD3, FOXP3, LAT, CTLA-4, T-bet, IL-2R alpha chain, Lck, PTPN22, IFN-gamma, CD4
**3**	Immune response_IL-16 signaling pathway	7.734E-11	IL-15, IL-2R beta chain, IL-16 mature, N-terminal IL16, FOXP3, cPKC (conventional), IL-2R alpha chain, CCR5, Lck, IL-16, CD4, PKC
**4**	Immune response_Inhibitory PD-1 signaling in T cells	1.068E-09	CD8 alpha, CD8, ZAP70, CD3, FOXP3, T-bet, PI3K cat class IA, CD3 zeta, Lck, CD4, FYB1

**5**	Role of Bregs in attenuation of T and NK cells mediated anti-tumor immune responses	1.144E-09	CD3, FOXP3, CTLA-4, IL-2R alpha chain, CD3 zeta, Adenosine A2a receptor, LAG3, Lck, FasL(TNFSF6), IFN-gamma
**6**	Chemotaxis_SDF-1/ CXCR4-induced chemotaxis of immune cells	7.955E-09	ZAP70, CD3, SFK, WASP, PLC-gamma 2, PI3K cat class IA, CD3 zeta, ITK, Lck, CD45, CALDAG-GEFI, PI3K cat class IB (p110-gamma)
**7**	Immune response_TCR alpha/beta signaling pathway	9.000E-09	CD8, ZAP70, CD3, P2X4, LAT, WASP, ITK, P2X1, Lck, CD45, CD4, FYB1, TRAF3
**8**	Immune response_Differentiation and clonal expansion of CD8+ T cells	1.351E-08	CD8, CD27(TNFRSF7), STAT4, IFN-alpha, T-bet, FasL(TNFSF6), Perforin, IFN-gamma, CD4
**9**	Immune response_T regulatory cell-mediated modulation of effector T cell and NK cell functions	2.425E-08	STAT4, FOXP3, T-bet, Granzyme A, IL-2R alpha chain, Adenosine A2a receptor, FasL(TNFSF6), Perforin, IFN-gamma, Adenylate cyclase type VII
**10**	Immune response_Role of DAP12 receptors in NK cells	2.425E-08	ZAP70, LAT, WASP, KIR2DL3, NKp44, PLC-gamma 2, PI3K cat class IA, KIR2DL1, KLRC3, Lck
**11**	Immune response_Immunological synapse formation	2.425E-08	ZAP70, CD3, LAT, Cytohesin1, WASP, PI3K cat class IA, ITK, CALDAG-GEFI, FYB1, PREL1
**12**	Dual function of Treg cells in cancer development	6.338E-08	FOXP3, CTLA-4, WNT, IL-2R alpha chain, FasL(TNFSF6), Perforin, IFN-gamma, CD45, CD4
**13**	SLE genetic marker-specific pathways in T cells	1.334E-07	ESR1 (nuclear), STAT4, ZAP70, CD3, LAT, IFN-alpha, T-bet, CD3 zeta, Ikaros, Lck, PTPN22, IFN-gamma
**14**	Immune response_NF-AT in immune response	1.626E-07	ZAP70, CD3, LAT, TRIM, PLC-gamma 2, PI3K cat class IA, CD3 zeta, ITK, Lck
**15**	Cell adhesion_Integrin inside-out signaling in T cells	1.938E-07	ZAP70, CD3, PIP5KI, CD3 zeta, Lck, CALDAG-GEFI, FYB1, CCR7, PREL1
**16**	Immune response_T cell subsets: cell surface markers	1.938E-07	CD161, CD8, CD3, CTLA-4, IL-2R alpha chain, LAG3, CCR5, CD4, CCR7
**17**	Immune response_CD16 signaling in NK cells	2.292E-07	ZAP70, LAT, Cytohesin1, WASP, PLC-gamma 2, PI3K cat class IA, CD3 zeta, Lck, FasL(TNFSF6), IFN-gamma
**18**	Aberrant production of IL-2 and IL-17 in SLE T cells	5.135E-07	CD229, ZAP70, CD3, LAT, SAP, CD3 zeta, Lck, CD3 epsilon, CD4

**19**	Role of CD8+ Tc1 cells in COPD	6.354E-07	IL-15, CD8, STAT4, T-bet, CXCR6, CCR5, Perforin, IFN-gamma
**20**	G-protein signaling_N-RAS regulation pathway	1.083E-06	ZAP70, CD3, LAT, CD3 zeta, Lck, CALDAG-GEFI, CD4
**21**	iNKT cell-keratinocyte interactions in allergic contact dermatitis	2.027E-06	CD161, CD3, T-bet, ITK, CD3 epsilon, FasL(TNFSF6), IFN-gamma
**22**	Chemotaxis_CXCR3-A signaling	2.325E-06	STAT4, ZAP70, CD3, LAT, T-bet, Lck, Perforin, IFN-gamma, PI3K cat class IB (p110-gamma)
**23**	Immune response_Regulation of T cell function by CTLA-4	2.464E-06	ZAP70, CD3, LAT, CTLA-4, PI3K cat class IA, CD3 zeta, Lck
**24**	T follicular helper cell dysfunction in SLE	2.828E-06	CD84, IRF4, SAP, TRIM, IFN-alpha, CTLA-4, PI3K cat class IA, BAFF-R, IFN-gamma, CD4
**25**	Immune response_IL-2 signaling via JAK/ STAT	3.022E-06	Cyclin D2, IL-2R beta chain, STAT4, FOXP3, IL-2R alpha chain, Perforin
**26**	Role of tumor-infiltrating B cells in anti-tumor immunity	3.130E-06	CD8, CD27(TNFRSF7), IRF4, IFN-alpha, T-bet, AID, FasL(TNFSF6), Perforin, IFN-gamma, CD4
**27**	CD8+ Tc1 cells in allergic contact dermatitis	3.873E-06	CD8 alpha, CD8, FasL(TNFSF6), Perforin, IFN-gamma, CD4
**28**	Immune response_IL-12 signaling pathway	4.214E-06	STAT4, CD3, T-bet, IL-2R alpha chain, PI3K cat class IA, CD3 zeta, Lck, Perforin, IFN-gamma
**29**	B cell signaling in hematological malignancies	5.886E-06	Cyclin D2, CD27(TNFRSF7), ZAP70, PLC-gamma 2, BAFF-R, PKC-beta2, PKC-beta1, PI3K cat class IA (p110-delta), TRAF3
**30**	Oxidative stress_Activation of NADPH oxidase	6.389E-06	cPKC (conventional), PI3K cat class IA, PKC-beta2, PLC-gamma, PI3K reg (p87-gamma), PI3K cat class IB (p110-gamma), p47-phox, PKC
**31**	CHDI_Correlations from Replication data_Causal network (positive correlations)	7.292E-06	ZAP70, CD3, LAT, PI3K cat class IA, CD83, ITK, Lck, CD45, PI3K cat class IB (p110-gamma)
**32**	COVID-19: immune dysregulation	7.383E-06	CD8, CD3, FOXP3, Granzyme A, CCR5, Perforin, IFN-gamma, sIL2RA, CD4, CCR7
**33**	Immune response_Role of DPP4 (CD26) in immune regulation	1.303E-05	ZAP70, CD3, CD3 zeta, Lck, FasL(TNFSF6), IFN-gamma, CD45

**34**	Dysregulation of germinal center response in SLE	1.333E-05	ESR1 (nuclear), IL-15, IRF4, IFN-alpha, AID, FasL(TNFSF6), PI3K cat class IA (p110-delta), CD21
**35**	Immune response_B cell antigen receptor (BCR) pathway	1.726E-05	Cyclin D2, HPK1(MAP4K1), PKC-beta, PIP5KI, WASP, PLC-gamma 2, PKC-beta2, PIP5KIII, PLC-gamma, PI3K cat class IA (p110-delta)
**36**	Rheumatoid arthritis (general schema)	1.983E-05	IL-15, FOXP3, IL-2R alpha chain, CD2, PTPN22, IFN-gamma, CD4
**37**	NK cells in allergic contact dermatitis	2.020E-05	NKp46, NKp44, CCR5, FasL(TNFSF6), Perforin, IFN-gamma
**38**	Immune response_OX40L/ OX40 signaling pathway	2.083E-05	CD3, FOXP3, T-bet, IL-2R alpha chain, PI3K cat class IA, Perforin, IFN-gamma, PLC-gamma
**39**	WNT signaling in gastric cancer	2.844E-05	WNT2B, WNT9A, WNT, WNT10B, IFN-gamma, WNT1
**40**	Immune response_T cell co-signaling receptors, schema	3.749E-05	CD27(TNFRSF7), DR3(TNFRSF12), CD30L (TNFSF8), CD244, CTLA-4, CD2, LAG3
**41**	Immune response_CD28 signaling	3.749E-05	ZAP70, CD3, LAT, PIP5KI, PI3K cat class IA, ITK, Lck
**42**	Role of iNKT and B cells in T cell recruitment in allergic contact dermatitis	4.566E-05	CD3, T-bet, AID, ITK, IFN-gamma, CD21
**43**	Memory CD8+ T cells in allergic contact dermatitis	5.296E-05	IL-15, IL-2R beta chain, CD8, T-bet, IFN-gamma, CCR7
**44**	Role of integrins in eosinophil degranulation in asthma	5.320E-05	CSF2RB, PI3K cat class IA, Plastin, PKC-beta2, IFN-gamma, PI3K cat class IB (p110-gamma), PKC
**45**	Generation of cytotoxic CD8+ T cells in COPD	5.425E-05	CD8, CTLA-4, Perforin, IFN-gamma, CD4
**46**	Immune response_ICOS signaling pathway in T-helper cell	6.640E-05	CD3, FOXP3, T-bet, PI3K cat class IA, ITK, IFN-gamma, CD4
**47**	Regulatory T cells in murine model of contact hypersensitivity	8.019E-05	CD8, CTLA-4, Adenosine A2a receptor, FasL(TNFSF6), CD4
**48**	CHDI_Correlations from Replication data_Cytoskeleton and adhesion module	1.009E-04	ZAP70, CD3, Cytohesin1, WASP, CALDAG-GEFI, PI3K cat class IB (p110-gamma), Collagen IV

**49**	Immune response_KLRK1 (NKG2D) signaling pathway	1.342E-04	ZAP70, PLC-gamma 2, PI3K cat class IA, Lck, IFN-gamma, DAP10
**50**	Chemokines in inflammation in adipose tissue and liver in obesity, type 2 diabetes and metabolic syndrome X	1.512E-04	CD8 alpha, EMR1, CD8, CD3, CCR5, CD45

Table S10. FABP12 differentially expressed genes pathway in CRC developed by MetaCore. Development_TGF-beta-dependent induction of EMT via MAPK was correlated with CRC development.

#	Maps	pValue	Network Objects from Active Data
**1**	Protein folding and maturation_Posttranslational processing of neuroendocrine peptides	6.916E-05	CCK8-Gly, CCK8-GlyArgArg, Pro-CCK, CCK8
**2**	Translation_Regulation of EIF2 activity	6.279E-04	DYRK2, DYRK1a, PKR
**3**	Development_TGF-beta-dependent induction of EMT via MAPK	1.173E-03	JNK(MAPK8-10), TGF-beta, TGF-beta 3
**4**	Apoptosis and survival_Role of PKR in stress-induced apoptosis	1.663E-03	Caspase-7, TRAM, PKR
**5**	Oxidative stress_Role of ASK1 under oxidative stress	1.756E-03	JNK(MAPK8-10), DYRK1a, JNK1(MAPK8)
**6**	Role of ER stress in obesity and type 2 diabetes	1.756E-03	PKR, HMGCS1, JNK1(MAPK8)
**7**	Tau pathology in Alzheimer disease	1.851E-03	JNK(MAPK8-10), DYRK1a, Caspase-7
**8**	Immune response_Role of PKR in stress-induced antiviral cell response	2.052E-03	JNK(MAPK8-10), Caspase-7, PKR
**9**	Cell adhesion_Desmosomes	3.159E-03	Desmocollin 3, DSC2
**10**	Ethanol/Acetaldehyde-dependent stimulation of MMP-9 expression in HCC	3.159E-03	JNK(MAPK8-10), JNK1(MAPK8)
**11**	Immune response_IFN-alpha/beta signaling via MAPKs	4.148E-03	JNK(MAPK8-10), PKR, JNK1(MAPK8)

**12**	The role of KEAP1/NRF2 pathway in skin sensitization	8.831E-03	JNK(MAPK8-10), JNK1(MAPK8)
**13**	Resistance of pancreatic cancer cells to death receptor signaling	9.374E-03	TRUNDD(TNFRSF10D), Caspase-7
**14**	Role of Apo-2L(TNFSF10) in Prostate Cancer cell apoptosis	9.932E-03	TRUNDD(TNFRSF10D), Caspase-7
**15**	Extracellular matrix-regulated proliferation of airway smooth muscle cells in asthma	1.050E-02	JNK(MAPK8-10), JNK1(MAPK8)
**16**	Production and activation of TGF-beta in airway smooth muscle cells	1.050E-02	TGF-beta, TGF-beta 3
**17**	Th2 cytokine- and TNF-alpha-induced inflammatory response in asthmatic airway fibroblasts	1.050E-02	JNK(MAPK8-10), Eotaxin-3
**18**	Development_TGF-beta-dependent induction of EMT via SMADs	1.050E-02	TGF-beta, TGF-beta 3
**19**	Immune response_IL-12 and IL-18-induced IFN-gamma production	1.109E-02	JNK(MAPK8-10), IL-12RB2
**20**	Transcription_CoREST complex-mediated epigenetic gene silencing	1.169E-02	ZNF516, CoREST
**21**	IFN-gamma and Th2 cytokines-induced inflammatory signaling in normal and asthmatic airway epithelium	1.358E-02	JNK(MAPK8-10), Eotaxin-3
**22**	Apoptosis and survival_Lymphotoxin-beta receptor signaling	1.490E-02	JNK(MAPK8-10), Caspase-7
**23**	Apoptosis and survival_TNFR1 signaling pathway	1.558E-02	JNK(MAPK8-10), Caspase-7
**24**	Role of platelets in allograft rejection	1.558E-02	P2Y1, TGF-beta
**25**	HGF receptor (Met) and MSP receptor (RON) signaling pathways in SCLC	1.558E-02	JNK(MAPK8-10), PKR
**26**	Role of TLR signaling in skin sensitization	1.628E-02	JNK(MAPK8-10), TRAM

**27**	TGF-beta 1-mediated induction of EMT in normal and asthmatic airway epithelium	1.628E-02	JNK(MAPK8-10), JNK1(MAPK8)
**28**	SCAP/SREBP Transcriptional Control of Cholesterol and FA Biosynthesis	1.699E-02	HMGCS2, HMGCS1
**29**	Immune response_Induction of apoptosis and inhibition of proliferation mediated by IFN-gamma	1.845E-02	Caspase-7, PKR
**30**	Role of proinflammatory cytokines in activation of p53 in major depressive disorder	1.845E-02	JNK(MAPK8-10), TGF-beta
**31**	Signal transduction_JNK pathway	1.845E-02	JNK(MAPK8-10), JNK1(MAPK8)
**32**	Stimulation of TGF-beta signaling in lung cancer	1.919E-02	TGF-beta, TGF-beta 3
**33**	Demyelination in multiple sclerosis	1.996E-02	JNK(MAPK8-10), JNK1(MAPK8)
**34**	Immune response_Lysophosphatidic acid signaling via NF-kB	2.231E-02	JNK(MAPK8-10), Caspase-7
**35**	Immune response_T cell subsets: cell surface markers	2.231E-02	IL-12RB2, CD94
**36**	Apoptosis and survival_Endoplasmic reticulum stress response pathway	2.562E-02	JNK(MAPK8-10), Caspase-7
**37**	Development_Keratinocyte differentiation	2.562E-02	MAD, JNK1(MAPK8)
**38**	CCR7 signaling pathways in dendritic cells in allergic contact dermatitis	2.648E-02	JNK(MAPK8-10), JNK1(MAPK8)
**39**	Cigarette smoke-induced oxidative stress and apoptosis in airway epithelial cells	2.912E-02	JNK(MAPK8-10), JNK1(MAPK8)
**40**	DNA damage_p53 activation by DNA damage	2.912E-02	JNK(MAPK8-10), DYRK2
**41**	TGF-beta-induced fibroblast/ myofibroblast migration and extracellular matrix production in asthmatic airways	2.912E-02	JNK(MAPK8-10), TGF-beta 3

**42**	Oxidative stress_ROS-mediated MAPK activation via canonical pathways	2.912E-02	JNK(MAPK8-10), JNK1(MAPK8)
**43**	Signal transduction_MIF signaling pathway	3.002E-02	JNK(MAPK8-10), SPPL2a
**44**	Effect of H. pylori infection on inflammation in gastric epithelial cells	3.002E-02	JNK(MAPK8-10), RIPK2
**45**	Immune response_IFN-alpha/beta signaling via JAK/STAT	3.093E-02	Caspase-7, PKR
**46**	Immune response_IL-18 signaling	3.186E-02	JNK(MAPK8-10), TRAM
**47**	Vascular endothelial cell damage in SLE	3.186E-02	JNK(MAPK8-10), Caspase-7
**48**	B-regulatory cells and tumor cells intercellular interaction	3.567E-02	JNK(MAPK8-10), IL-12RB2
**49**	Eosinophil survival in asthma	3.567E-02	TRUNDD(TNFRSF10D), Eotaxin-3
**50**	Mucin expression in CF airways	3.763E-02	JNK(MAPK8-10), JNK1(MAPK8)
